# The function of LHCBM4/6/8 antenna proteins in *Chlamydomonas reinhardtii*

**DOI:** 10.1093/jxb/erw462

**Published:** 2016-12-22

**Authors:** Laura Girolomoni, Paola Ferrante, Silvia Berteotti, Giovanni Giuliano, Roberto Bassi, Matteo Ballottari

**Affiliations:** 1 Dipartimento di Biotecnologie, Università di Verona, Strada le Grazie 15, 37134, Verona, Italy; 2 Italian National Agency for New Technologies, Energy and Sustainable Development (ENEA), Casaccia Research Center, Rome, Italy

**Keywords:** Chloroplast, gene silencing, microalgae, photoprotection, photosynthesis, photosystems, state transitions.

## Abstract

In eukaryotic autotrophs, photosystems are composed of a core moiety, hosting charge separation and electron transport reactions, and an antenna system, enhancing light harvesting and photoprotection. In *Chlamydomonas reinhardtii*, the major antenna of PSII is a heterogeneous trimeric complex made up of LHCBM1–LHCBM9 subunits. Despite high similarity, specific functions have been reported for several members including LHCBM1, 2, 7, and 9. In this work, we analyzed the function of *LHCBM4* and *LHCBM6* gene products *in vitro* by synthesizing recombinant apoproteins from individual sequences and refolding them with pigments. Additionally, we characterized knock-down strains *in vivo* for *LHCBM4/6/8* genes. We show that LHCBM4/6/8 subunits could be found as a component of PSII supercomplexes with different sizes, although the largest pool was free in the membranes and poorly connected to PSII. Impaired accumulation of LHCBM4/6/8 caused a decreased LHCII content per PSII and a reduction in the amplitude of state 1–state 2 transitions. In addition, the reduction of LHCBM4/6/8 subunits caused a significant reduction of the Non-photochemical quenching activity and in the level of photoprotection.

## Highlight

LHCBM4/6/8 are three closely related light-harvesting proteins in *Chlamydomonas reinhardtii*. Reverse genetics show that they have specific functions in photoprotection, namely in non-photochemical quenching and state transitions.

## Introduction

Life on Earth is fueled by photon energy harvested by photosynthetic systems. In green algae and land plants, photosynthesis occurs in chloroplasts, where two pigment-binding protein complexes, PSI and PSII, catalyze the light-dependent steps of electron transport from water to NADP^+^ which is coupled to proton transport to the thylakoid lumen for ATP synthesis. Each photosystem includes two moieties: a core complex binding electron transport cofactors and a peripheral antenna system enhancing the cross-section and providing photoprotection. The PSII core complex is highly conserved in all photosynthetic organisms and is comprised of the chlorophyll-binding subunits D1 and D2, the Chl *a*-binding antenna complexes CP43 and CP47, and the cytochrome *b*_559_. The outer antenna system of PSII is made up of pigment-binding light-harvesting complexes called LHCII (light-harvesting complex II), a trimeric complex made by 22–26 kDa polypeptides with three transmembrane and two amphipatic α-helices exposed to the lumen (Kühlbrandt *et al.*, 1994; Liu *et al.*, 2004; Standfuss *et al.*, 2005), each binding up to 14 chlorophylls and four xanthophylls. These chromophores are bound to multiple specific sites for xanthophylls (L1, L2, N1, and V1) as well as for chlorophylls (Chl601-614) (Croce *et al.*, 1999*a*, *b*; Caffarri *et al.*, 2001, 2004, 2007; Liu *et al.*, 2004; Ballottari *et al.*, 2012). LHC proteins harvest light energy and transfer excitons to the core complexes. LHCs also have a crucial role in photoprotection (Havaux and Tardy, 1997; Elrad *et al.*, 2002; Ballottari *et al.*, 2012; Dall’Osto *et al.*, 2010; Grewe *et al.*, 2014), provided by their carotenoid ligands, namely lutein, neoxanthin, and violaxanthin, which are involved in quenching chlorophyll triplet excited states and reactive oxygen species (ROS) scavenging (Dall’Osto *et al.*, 2006, 2007, 2013; Li *et al.*, 2009; Ballottari *et al.*, 2012, 2013). In high light conditions, when absorbed energy exceeds the capacity of downstream metabolic reactions, photoprotection is enhanced by synthesis of zeaxanthin, which replaces violaxanthin (Havaux *et al.*, 2007; Ahn *et al.*, 2008; Dall’Osto *et al.*, 2010). Furthermore, LHC proteins are involved in fast regulative responses to unbalanced excitation of PSI versus PSII in limiting light, namely state 1–state 2 transitions (Allen and Pfannschmidt, 2000; Finazzi *et al.*, 2002; Depège *et al.*, 2003; Ferrante *et al.*, 2012; Galka *et al.*, 2012; Allorent *et al.*, 2013; Benson *et al.*, 2015) and in non-photochemical quenching of excitation energy (NPQ) (Elrad *et al.*, 2002; Ruban *et al.*, 2007; Peers *et al.*, 2009; de Bianchi *et al.*, 2011; Betterle *et al.*, 2015) in excess light. Optimal use of limiting light is obtained by balancing PSII and PSI antenna sizes by transferring a subset of the LHCII from PSII to PSI whenever plastoquinone is over-reduced. Over-reduction of the plastoquinone pool activates a kinase (STT7) phosphorylating LHCII and favoring its migration to PSI (Allen and Pfannschmidt, 2000; Finazzi *et al.*, 2002; Depège *et al.*, 2003; Ferrante *et al.*, 2012; Galka *et al.*, 2012; Allorent *et al.*, 2013; Drop *et al.*, 2014*a*, *b*; Ünlü *et al.*, 2014; Benson *et al.*, 2015; Nawrocki *et al.*, 2016). In *Chlamydomonas reinhardtii*, trimeric LHCII is encoded by nine genes called *LHCBM1–LHCBM9*, with M referring to ‘major’ antenna complex (Merchant *et al.*, 2007; Ferrante *et al.*, 2012). The *LHCBM4*, *6*, *8*, and *9* genes are localized on chromosome 6, *LHCBM2* and *7* on chromosome 12, and *LHCBM5* on chromosome 3, whereas the isoforms *LHCBM1* and *LHCBM3* have not yet been mapped (Drop *et al.*, 2014*a*). *LHCBM* gene products have sequence identity of ~70% and cluster into four groups: Type I (LHCBM3, LHCBM4, LHCBM6, LHCBM8, and LHCBM9), Type II (LHCBM5), Type III (LHCBM2 and LHCBM7), and Type IV (LHCBM1) (Drop *et al.*, 2014*a*), with members of the same subgroup showing identity up to 99% (Natali and Croce, 2015). Knowledge of LHCII structure and function is based on the orthologous complexes from higher plants. Common features include amino acid ligands for chlorophylls, the lumen-exposed tyrosine residue, essential for binding neoxanthin, and the N-terminal domain exposed to the chloroplast stroma which mediates interactions such as trimerization (Hobe *et al.*, 1995; Natali and Croce, 2015). Despite their high similarity, LHCBM components are functionally specialized: reverse genetics applied to LHCBM2/7and LHCBM5 (Takahashi *et al.*, 2006; Ferrante *et al.*, 2012) suggest that they are involved in state 1–state 2 transitions, while LHCBM1 (Elrad *et al.*, 2002) plays an important role in thermal energy dissipation probably as an interactor of LHCSR3, the trigger for NPQ (Peers *et al.*, 2009; Bonente *et al.*, 2011). A special case is LHCBM9, which is preferentially expressed in nutrient starvation or anaerobiosis (Nguyen *et al.*, 2008) to provide protection for PSII (Grewe *et al.*, 2014). Structural analysis suggests that LHCBM1, LHCBM2, and LHCBM3 participate in PSII supercomplexes while LHCBM5 belongs to the ‘extra’ LHCII pool more loosely associated with the core complexes (Drop *et al.*, 2014*a*). Here, we have studied the role of the LHCBM4, LHCBM6, and LHCBM8 proteins by using artificial microRNA (amiRNA) silencing to silence co-ordinately gene subfamilies sharing identical regions, while keeping the level of expression of others unaltered (Molnar *et al.*, 2009; Zhao *et al.*, 2009; Ferrante *et al.*, 2012; Grewe *et al.*, 2014). The phenotypic analysis was complemented by studying biochemical and spectroscopic proteins of pigment–protein subunits obtained by refolding *in vitro* the apoproteins expressed in bacteria, to yield a comprehensive explanation of the function of these three LHC subunits in *C. reinhardtii*.

## Materials and methods

### Strains and culture conditions

Unless indicated differently, *C. reinhardtii* cells were grown at 25 °C with fluorescent white light (60 μE m^−2^ s^−1^) with a 16 h light:8 h dark photoperiod in HS medium. The cell wall-less *cw15* strain was transformed with the recombinant pChlamyRNA3 vectors (Molnar *et al.*, 2009) containing the amiRNAs for silencing of *LHCBM6* or *LHCBM4*, *LHCBM6* and *LHCBM8*. Nuclear transformation was performed as described (Kindle, 1990). Transformants were selected on TAP agar plates containing paromomycin (10 μg ml^−1^) as previously described (Ferrante *et al.*, 2012). To screen the silenced LHCBM6 and LHCBM4+6+8 transformants based on Chl *a*/*b* ratios, cells were grown in 96-well microtiter plates in 200 μl of TAP at 25 °C until the stationary phase (2 × 10 ^7^ cells ml^−1^) with fluorescent white light (60 μE m^−2^ s^−1^) with a 16 h light:8 h dark photoperiod. Ninety transformants were analyzed for each construct. Chl *a*/*b* ratios were determined on pigment extracts as described in Ferrante *et al.* (2012). To perform quantitative real-time PCR, transformants showing increased Chl *a*/*b* ratios were grown in 4 ml of TAP medium in 24-well microtiter plates until the late-log phase with fluorescent white light (60 μE m^−2^ s^−1^) with a 16 h light:8 h dark photoperiod, and cells were harvested for RNA extraction.

### Plasmid construction and quantitative real-time RT-PCR

amiRNAs used to silence *LHCBM* genes were designed using the WMD3 software (Web micro RNA designer Version3, http://wmd3.weigelworld.org/cgi-bin/webapp.cgi?page=Home;project=stdwmd) and verified using the EST database (http://est.kazusa.or.jp/en/plant/chlamy/EST/blast.html). Two amiRNAs were designed for silencing of the *LHCBM6* gene, the former (LHCBM6A) annealing in the 3'-untranslated region (UTR) and the latter (LHCBM6B) annealing in the 5'-UTR of the gene. Cloning of the amiRNAs in the pChlamyRNA3 vector, total RNA extraction from *Chlamydomonas* transformants, and real-time RT-PCR were performed as previously described (Ferrante *et al.*, 2012). In particular, cells were harvested for RNA extraction in the light period after 6 h of light. Oligonucleotides used for RT-PCR are reported in Supplementary Table S1 at *JXB* online.

### *Protein purification and* in vitro *reconstitution*

LHCBM4 and LHCBM6 coding sequence for the mature proteins were cloned in the pET28 expression vector and overexpressed in *Escherichia coli*. The signal peptide sequence was identified as described in the literature (Turkina *et al.*, 2006). Inclusion bodies we purified as previously described (Giuffra *et al.*, 1996) and *in vitro* refolding upon addition of pigments was performed as previously reported (Giuffra *et al.*, 1996; Grewe *et al.*, 2014).

### Pigment analysis

Pigment analysis were performed by HPLC as described in Lagarde *et al.* (2000). Chl *a/b* and chlorophyll/carotenoid ratios were corrected through fitting analysis of the absorption spectrum (Croce *et al.*, 2002).

### *Thylakoid preparation from* C. reinhardtii *cells*

*Chlamydomonas reinhardtii* stacked thylakoids were purified as described in Ferrante *et al.* (2012).

### SDS–PAGE and immunoblotting

Denaturing SDS–PAGE was performed in the presence of 6 M urea with the Tris-Tricine buffer systems (Schägger and von Jagow, 1987). Immunoblotting analyses were performed using α-CP43, α-PsaA, and α-LHCBM5 (herein renamed α-LHCII) from Agrisera and using α-LHCSR3 described in Bonente *et al.* (2012) and α-LHCBM6 described in Berger *et al.* (2014).

### Native electrophoresis

Thylakoid membranes were solubilized in the presence of 1.2% α-dodecyl-maltoside and separated by Clear Native (CN)–PAGE as described in Grewe *et al.* (2014).

### PSI and PSII functional antenna size

Relative PSI antenna size was estimated from kinetics of P700 oxidation in limiting orange light (12 μE m^−2^ s^−1^) in thylakoids treated with DCMU [3-(3,4-dichlorophenyl)-1,1-dimethylurea], ascorbate, and methyl viologen, as described in Bonente *et al.* (2012). In particular, the P700 oxidation kinetics were fitted with exponential functions and the reciprocal of rate constants extrapolated were used to estimate the PSI antenna size (Bonente *et al.*, 2012). PSII antenna size has been estimated in whole cells from *F*_m_ saturation kinetics (1/τ_2/3_) in the presence of 10^−5^ M DCMU (Cardol *et al.*, 2008).

### State transitions

The amplitude of state 1–state 2 transition was investigated by two approaches: (i) LHCII detachment from PSII upon state 2 induction was followed by measuring the differences in the maximal fluorescence emitted by PSII in state 1 or state 2 conditions as previously described (Bonente *et al.*, 2012; Ferrante *et al.*, 2012; Fleischmann *et al.*, 1999). The second method (ii) consisted of measuring the 77K fluorescence emission spectra of whole cells in state 1 or state 2 conditions: the extent of induction of state transitions was expressed as the ratio between the peaks of PSI in state 2/state 1, prior to normalization to the peak of PSII in the two different conditions, respectively.

### NPQ measurements

NPQ measurements were performed on cells acclimated to high light conditions (400 μE m^−2^ s^−1^) at exponential growth phase. Cells were pre-illuminated for 2 min with a weak (3 μE m^−2^ s^−1^) far-red light-emitting diode (LED) before NPQ analysis with a PAM-101 (Waltz, Effeltrich, Germany); actinic light was 1600 μE m^−2^ s^−1^ and saturating light 4080 μE m^−2^ s^−1^. The far-red LED was kept on during dark recovery.

### Singlet oxygen production

Singlet oxygen production was measured *in vivo* by following the 532 nm fluorescence emission of a Singlet Oxygen Sensor Green (SOSG) probe (Flors *et al.*, 2006).

## Results

### In vitro *study of LHCBM4/6/8 proteins*

**Fig. 1.**
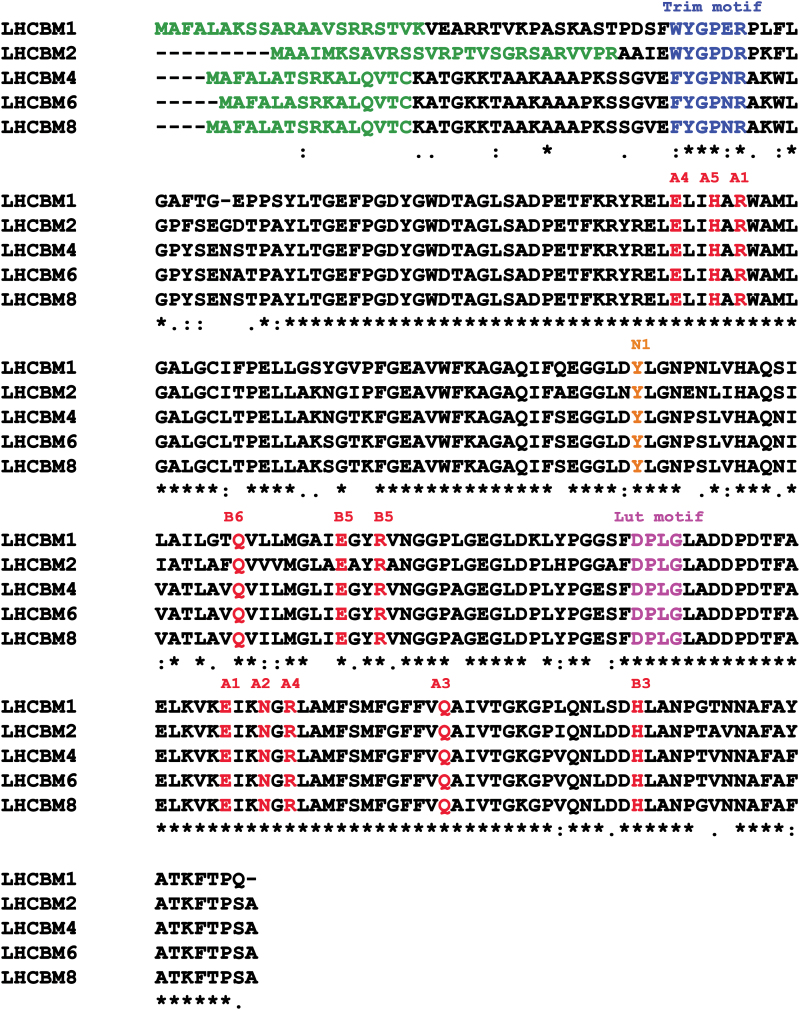
Alignment of LHCBM1, LHCBM2, LHCBM4, LHCBM6, and LHCBM8 polypeptide sequences. The signal peptide is indicated in green, the trimerization motif in blue, chlorophyll-binding sites in red, the lutein-binding motif in purple, and the tyrosine responsible for neoxanthin binding in the N1 site is indicated in orange.

**Fig. 2.**
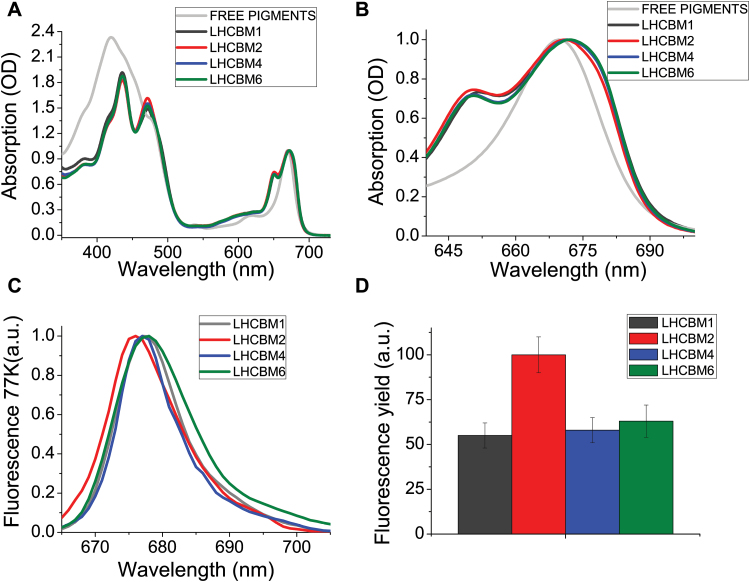
Absorption spectra and fluorescence yield of LHCBM1, LHCBM2, LHCBM4, and LHCBM6 recombinant proteins. (A) Absorption spectra in the 350–750 nm range normalized to the maximum peak in the Qy region. (B) Absorption spectra of LHCBM complexes zoomed in the 630–700 nm range. (C) 77K fluorescence emission spectra of LHCBM complexes upon excitation at 440 nm. (D) Relative fluorescence quantum yield of LHCBM1, LHCBM4, and LHCBM6 compared with LHCBM2, set to 100%. SDs are reported for each sample (*n*=5).

*LHCBM4*, *LHCBM6*, and *LHCBM8* genes are paralogous, with a high level of identity to each other (Ferrante *et al.*, 2012). The protein sequences of LHCBM4 (XP_001695344.1), LHCBM6 (XP_001695353.1), and LHCBM8 (XP_001695467.1) are characterized by an identity of 97.63%, with only three substitutions in their amino acid sequence (Fig. 1), and one deletion in the case of LHCBM6 localized in the first 26 residues constituting the transit peptide for chloroplast import. Alignment of LHCBM4, LHCBM6, and LHCBM8 sequences with LHCBM1 and LHCBM2 suggested that all the residues involved in chlorophyll and neoxanthin binding at the N1 site were conserved (Liu *et al.*, 2004; Caffarri *et al.*, 2007). Also, the DPLG motif which was previously associated with lutein binding (Kühlbrandt and Wang, 1991) is conserved in all the subunits herein considered. The trimerization motif WYxxxR was conserved in LHCBM1 and LHCBM2 but not in LHCBM4, 6, and 8 due to replacement of W by F. The LHCBM4 and LHCBM6 apoproteins were produced by expressing the gene sequences in *E. coli*, and holocomplexes were obtained by in *vitro* refolding with pigments (Giuffra *et al.*, 1996). The absorption spectra of both holoproteins showed a red shift of the Qy transition compared with free pigments in detergent solution (Fig. 2) while the Chl *b* → Chl *a* energy transfer efficiency was high as measured from overlapping fluorescence emission spectra with different excitation, namely 440, 475, and 500 nm for Chl *a*, *b*, or carotenoids (Supplementary Fig. S1), suggesting a correct folding of the protein–pigment complex (Giuffra *et al.*, 1996). The fluorescence emission spectra at 77K of the LHCBM proteins revealed significant differences: LHCBM2 emission was blue-shifted, with an emission peak at 677 nm, while LHCBM1 and LHCBM4 showed an intermediate behavior and LHCBM6 showed the red-most shifted subunit with a peak at 679 nm. ‘Red’ emission forms are associated with chlorophyll ligands with low energy transitions. We thus proceeded to assess the relative fluorescence quantum yield of the reconstituted LHCBM4 and LHCBM6 pigment–proteins. We used as a reference LHCBM1 and LHCBM2 subunits previously characterized as the gene products with, respectively, the lowest and the highest fluorescence quantum yield (Fig. 2) (Grewe *et al.*, 2014; Natali and Croce, 2015). LHCBM4 and LHCBM6 showed an intermediate fluorescence yield, more similar to LHCBM1 than to LHCBM2. This result suggests a similar role for LHCBM4, LHCBM6, and LHCBM1 in defining the lifetime of the excited states of the antenna system. Pigment analysis of reconstituted proteins showed a Chl *a/b* molar ratio ranging between 1.1 and 1.4, while the number of xanthophylls ranged from three to four based on 14 chlorophylls bound by each subunit (Liu *et al.*, 2004; Grewe *et al.*, 2014; Natali and Croce, 2015). The number of lutein ligands varied from 1.21 to 1.81, violaxanthin was substoichiometric (0.06–0.28), and neoxanthin ranged between 1.5 and 2.19. On this basis, it can be inferred that all LHCBM proteins analyzed bind lutein in the L1 site, as previously reported for LHCII (Liu *et al.*, 2004), while the L2 site can be occupied by lutein, violaxanthin, or neoxanthin, as previously reported for monomeric Lhcb subunits from higher plants (Ballottari *et al.*, 2009; Pan *et al.*, 2011). Neoxanthin is probably bound to the N1 site, while the most peripheral site, V1, can be partially occupied by violaxanthin or by neoxanthin according to previous suggestions (Caffarri *et al.*, 2007; Natali and Croce, 2015). The high sequence similarity between LHCBM8 and LHCBM4 (Fig. 1) suggests that conclusions drawn for LHCBM4 and LHCBM6 might also hold true for LHCBM8.

### LHCBM4/6/8 accumulation in thylakoid membranes

Accumulation of LHCBM4/6/8 in thylakoid membranes in *C. reinhardtii* was investigated by immunoblotting using recombinant proteins refolded *in vitro* as standards. Immunoblot analysis was performed on thylakoid membranes purified from the *C. reinhardtii* wild-type strain using an antibody recognizing all LHCBM proteins (α-LHCII) and a specific antibody for LHCBM4/6/8 subunits (Berger *et al.*, 2014). α-LHCBM4/6/8 antibody was tested for cross-reactivity with other LHCBM proteins, revealing only a minor cross-reaction against LHCBM3 and LHCBM9, with signals respectively 16-, 40-, and 18-fold weaker compared with LHCBM4 and LHCBM6 (Supplementary Fig. S2). Immunoblotting reactions on thylakoid membranes using the α-LHCII antibody yielded three main bands with apparent mol. wts of ~26, ~23, and ~22 kDa (Ferrante *et al.*, 2012) (Supplementary Fig. S3). LHCBM1 was reported to be the only gene product in the band with intermediate mobility, LHCBM2 and LHCBM7 were reported to migrate with the most mobile band (Ferrante *et al.*, 2012), while LHCBM9 migrated with the upper band (Grewe *et al.*, 2014). Using the α-LHCBM6 antibody yielded a single band, with mobility corresponding to the LHCBM band with the highest apparent molecular weight. Recombinant LHCBM4 and LHCBM6 were recognized by α-LHCBM6 antibody with a slightly higher apparent molecular weight compared with the native LHCBM4/6/8 subunits in thylakoid membranes. The same behavior was observed in the case of recombinant LHCBM1 compared with the native LHCBM1: this is likely to be related to the presence of extra amino acids at the N-terminus in the recombinant proteins, part of the chloroplast transit peptide which are cleaved in the mature native proteins. By using recombinant LHCBM proteins and native LHCII trimers as standards it was possible to determine that LHCBM4/6/8 are present in the thylakoid membranes with a similar abundance to LHCBM1, contributing to ~30% of the total pool of LHCII (Supplementary Fig. S3).

### *Silencing of* LHCBM *genes*

**Fig. 3.**
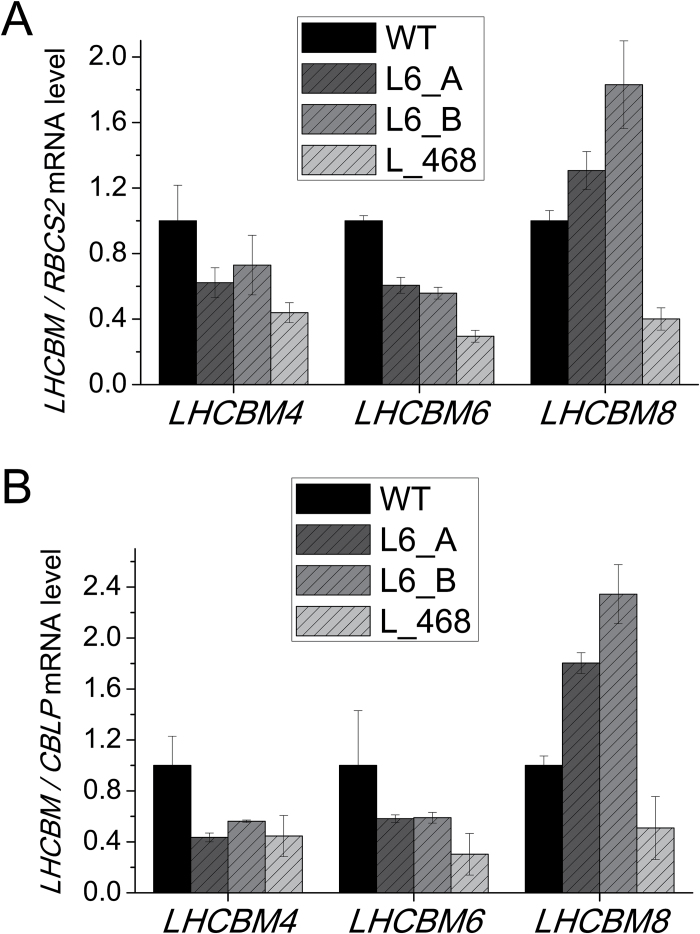
Quantification of *LHCBM* mRNA levels in knock-down strains. *LHCBM4*, *6*, and *8* mRNA abundance was quantified through quantitative real-time RT-PCR. The amounts of *LHCBM* mRNA are expressed using as reference the ribulose bisphosphate carboxylase/oxygenase small subunit 2 (*RBCS2*) mRNA level. Two different transformants silenced in the *LHCBM6* gene (L6_A and L6_B) and one transformant silenced in *LHCBM4*, *LHCBM6*, and *LHCBM8* genes were analyzed (L_468).

*Chlamydomonas* strains with reduced level of LHCBM4, LHCBM6, and LHCBM8 subunits were produced by amiRNA silencing according to previous reports (Molnar *et al.*, 2009; Ferrante *et al.*, 2012; Grewe *et al.*, 2014). Two amiRNAs were designed to silence the *LHCBM6* gene (Supplementary Table S2; Supplementary Fig. S4), while four different amiRNAs were selected for the simultaneous silencing of *LHCBM4*, *LHCBM6*, and *LHCBM8*, but only one (Supplementary Table S2; Supplementary Fig. S4) was effective in triggering silencing of this subgroup of genes (Supplementary Fig. S4). The designed amiRNAs were expressed under the control of the *PSAD* constitutive promoter in the *cw15* strain (referred to as the as the wild type in the following) and 90 transformants for each construct were screened based on their absorption spectra for Chl *a/b* ratios and confirmed by HPLC: since Chl *b* is bound to LHC proteins only while Chl *a* is bound to both LHC and core complexes, an increased Chl *a/b* ratio is a good indicator of reduced LHC protein content. A selection of transformants (~10 per construct) showing an increased Chl *a/b* ratio were investigated by real-time PCR, in order to confirm the silencing of the target genes. From this analysis, we selected the transformants showing the highest level of silencing: clones L6_A and L6_B (Supplementary Table S2; Supplementary Fig, S5). As shown in Fig. 3, the L6_A and L6_B transformants showed an ~40% decrease in *LHCBM6* mRNA level and a concomitant decrease of the *LHCBM4* mRNA level, while the *LHCBM8* gene in these strains was overexpressed as compared with the wild type. The increased expression of the *LHCBM8* gene when *LHCBM4* and *LHCBM6* are down-regulated suggests that the functions of these three subunits are redundant and that LHCBM8 probably accumulates in order to compensate for the reduction in LHCBM4 and LHCBM6. The L_468 transformant shows instead a decrease of ~65, ~70, and ~50–60%, respectively, in the level of *LHCBM4*, *LHCBM6*, and *LHCBM8* mRNAs. In order to evaluate the levels of off-target silencing, the expression level of all *LHCBM* genes was evaluated (Supplementary Fig. S4). Some off-target silencing was found for *LHCBM3* in the L6_A transformant and for *LHCBM7* and LHCBM5 in the L6_B transformant, while the L_468 transformant did not show statistically significant off-target silencing. The off-target effects were different in the different strains and were disregarded in the case of a consistent phenotype among the analyzed strains (Supplementary Fig. S5). Interestingly, in the strain with a lower expression of *LHCBM4*, *LHCBM6*, and *LHCBM8* genes, namely the L_468 strain, an increased *LHCBM1* and *LHCBM9* expression was detected (Supplementary Fig. S5).

### Photosynthetic protein abundance in knock-down strains

**Fig. 4.**
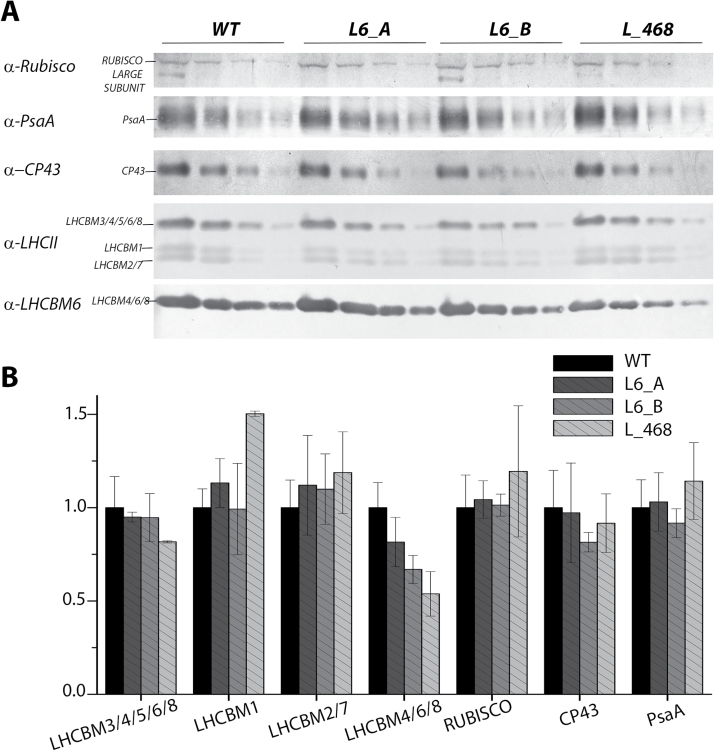
Immunoblot analysis of photosynthetic proteins in knock-down strains. Immunoblot analysis was performed using a specific antibody for PSI (α-PsaA), PSII (α-CP43), LHCs (α-LHCII), and LHCBM6 (α-LHCBM6). Three different sample amounts were loaded based on the chlorophyll content (0.9, 0.3, and 0.015 µg of chlorophyll). Densitometric quantification of each band normalized to the wild type is reported in (B). SDs are reported for each quantification (*n*=4).

**Fig. 5.**
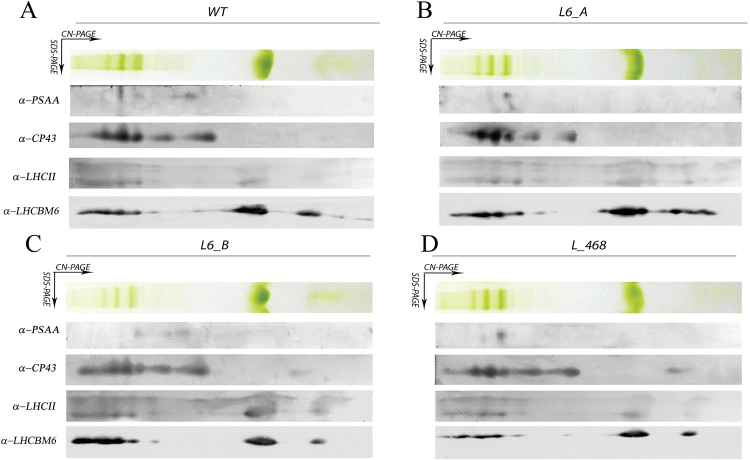
Analysis of the thylakoid membrane pigment–protein complexes by 2D electrophoresis and immunoblotting. Thylakoid membranes of knock-down strains grown in control light were solubilized with 1% dodecyl-maltoside (α-DM) and separated by CN–PAGE followed by a second dimension separation by SDS–PAGE. Immunoblot detection of LHCBM4/6/8, LHCII, PSI (antibody α-PSAA), and PSII (antibody α-CP43) is also reported. (This figure is available in colour at *JXB* online.)

Knock-down strains were analyzed by western blotting in order to evaluate the accumulation of LHCBM protein(s) compared with the wild type. All knock-down mutants showed a decrease in LHCBM6/4/8 content per chlorophyll as compared with the wild type, especially in the case of the L_468 strain. As reported in Fig. 4B, the accumulation of the different bands recognized by the α-LHCII antibody were similar in all cases, with the exception of L_468 where an increased accumulation of LHCBM1 was accompanied by a reduction of the signal at the higher apparent molecular weight, consistent with the strong reduction LHCBM4/6/8 subunits revealed by the α-LHCBM6 antibody (Fig. 4). Partial compensation of LHCBM4/6/8 reduction by accumulation of LHCBM1 is consistent with the transcript analysis reported in Fig. 3. Knock-down strains were characterized by a similar accumulation of CP43 and PsaA per chlorophyll compared with the wild type, The amount of Rubisco was also investigated, as an indicator for the accumulation of Calvin–Benson cycle enzymes in the transformants compared with the wild type, yielding a similar Rubisco/chlorophyll ratio in wild-type and knock-down strains. The organization of photosynthetic pigment–proteins was evaluated by 2D electrophoresis of solubilized thylakoid membranes on non-denaturing CN–PAGE as the first dimension while the second dimension was SDS–PAGE (Grewe *et al.*, 2014) (Fig. 5). Distinct subunits of the protein complexes were detected after 2D electrophoresis by immunoblotting with specific antibodies against PsaA (sbunit of PSI), CP43 (subunit of PSII), LHCII, and LHCBM4/6/8 (Fig. 5). The chlorophyll distribution in the CN–PAGE and the levels of immunoblot signals were quantified by densitometry and reported in Supplementary Fig. S6, for the wild type. PSII and PSI complexes were resolved at a high apparent molecular weight in CN–PAGE as PSI(I)–core, or as supercomplexes binding different amounts of LHC subunits. LHCII subunits could be found as monomers, trimers, or in supercomplexes, together with PSI or PSII subunits (Fig. 5; Supplementary Fig. S6). In the wild type, LHCBM4/6/8 subunit distribution was similar to other LHCII subunits and yet the intensity of the signal corresponding to trimers and monomers was clearly higher than that corresponding to PSII supercomplexes (Supplementary Fig. S6). The pattern of PsaA, CP43, LHCII, and LHCBM6 was not significantly altered in knock-down strains compared with the wild type, except for a reduced intensity of LHCBM4/6/8, as expected (Fig. 5). This result suggests that LHCBM4/6/8 could be preferentially found as free LHCII trimers, even if a minor fraction of these subunits was associated with PSI and PSII supercomplexes of different size.

### Roles of LHCBM4/6/8 in light harvesting and photoprotection

**Fig. 6.**
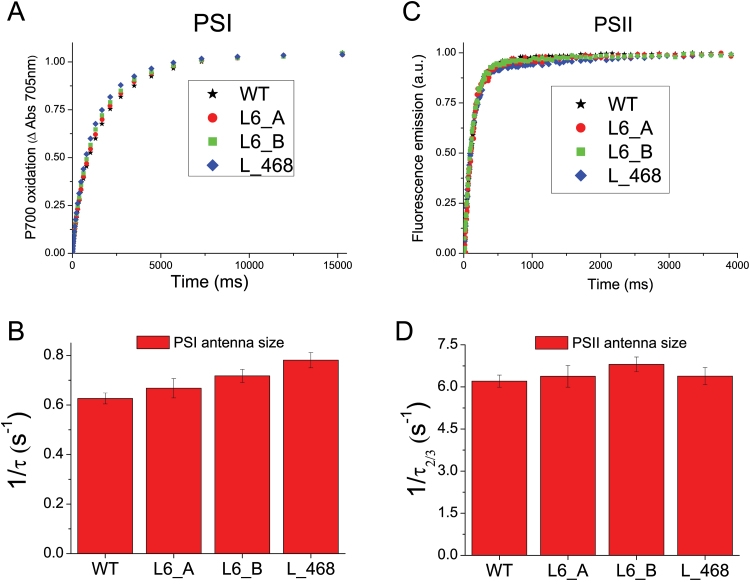
PSI and PSII antenna size measurements. PSI antenna size (A) was measured in wild-type (WT) and knock-down strains by following the kinetics of P700 oxidation in limiting light conditions in DCMU-, ascorbate-, and methyl viologen-treated thylakoids. P700 oxidation kinetics were fitted with exponential functions, and the reciprocal of time constants associated with fitting functions are reported in (B) normalized to the WT as an estimate of PSI antenna size. PSII antenna size (C) was measured by following the fluorescence emission kinetics of PSII in DCMU-treated cells. Fluorescence kinetics were fitted with exponential functions by which τ_2/3_ was calculated as the time required to reach two-thirds of the maximum fluorescence emission: the reciprocal of τ_2/3_ is plotted in (D) as an estimation of PSII antenna size. Data reported in (B) and (D) were tested for their statistical significance compared with the WT by Student *t*-test (*n*=3), obtaining in all cases *P*-values >0.05, indicating that the differences observed were not statistically significant.

**Fig. 7.**
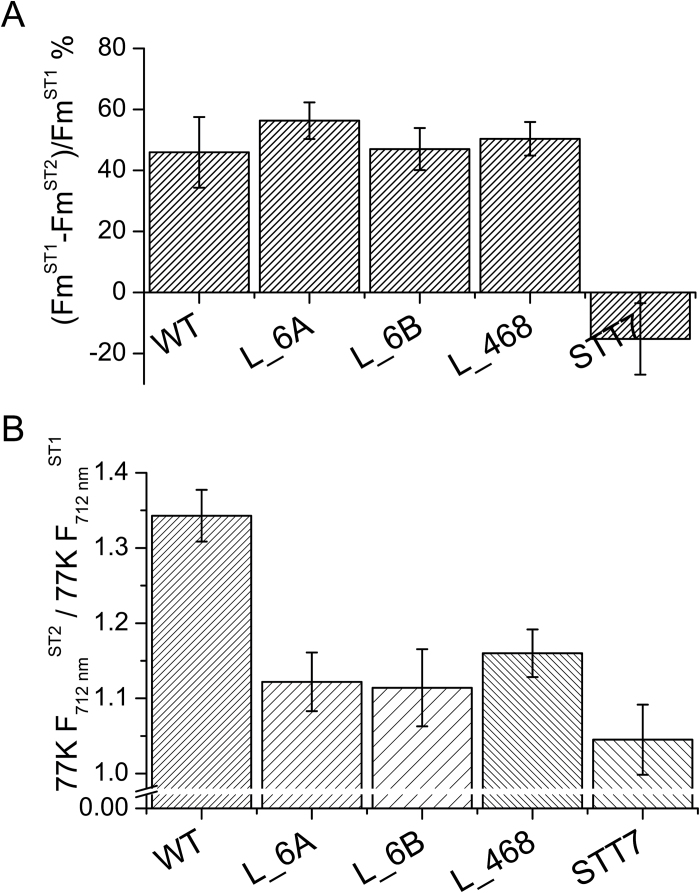
State 1–state 2 transition analysis. (A) Maximal capacity of switching LHCII antenna from PSII to PSI was analyzed in wild-type (WT) and knock-down strains by measuring the variation in maximum fluorescence emission in state 1 (*F*_m_^ST1^) and state 2 (*F*_m_^ST2^) at room temperature. The changes in *F*_m_ are related to PSII fluorescence emission. (B) Fluorescence emission spectra of cells in state 1 or state 2 were measured at 77K, the spectra were normalized to PSII peaks (686 nm), and the ratio between the PSI peaks (712 nm) in state 2 and state 1 is reported as 77K *F*_712_ nm^ST2^/77K *F*_712_ nm^ST1^. The changes in 712 nm fluorescence emission are related to PSI. In both panels, the *stt7* mutant was used as the negative control. Error bars indicate the SD (*n*=3).

**Fig. 8.**
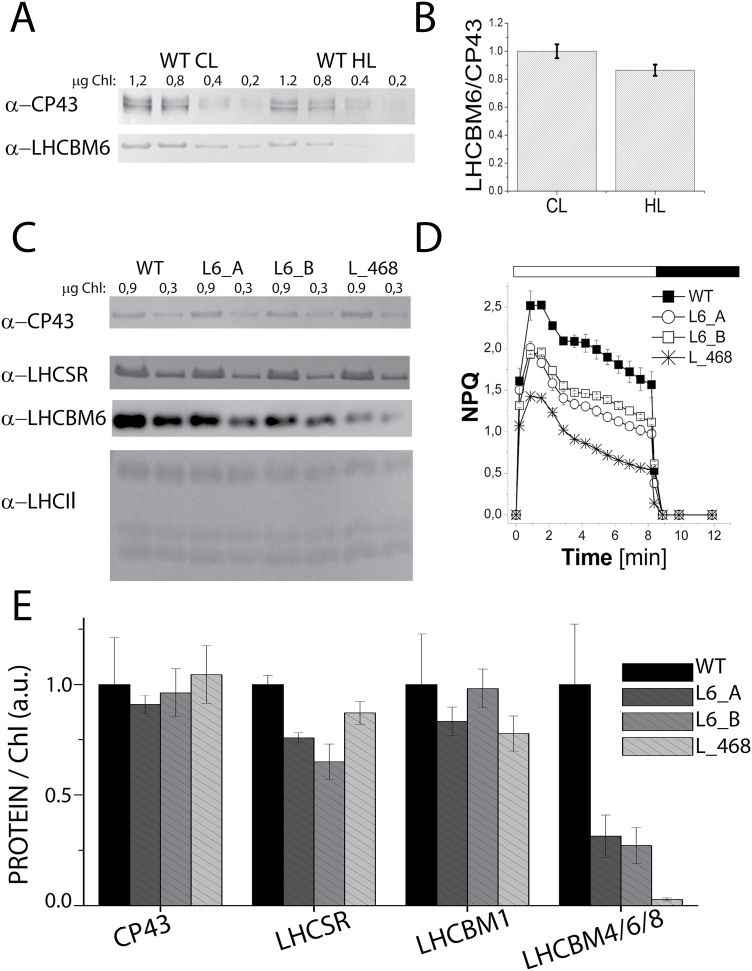
LHCBM4/6/8 accumulation and non-photochemical quenching (NPQ) induction in high light. Accumulation of LHCBM4/6/8 in high light (HL) compared with control light (CL) was analyzed by western blot (A; chlorophyll loading in each lane is reported on the top of the figure) and estimated upon normalization to CP43 content (B). The NPQ induction kinetics were collected by using an actinic light of 1500 µmol photons m^−2^ s^−1^ on HL-acclimated cells (C). Accumulation of LHCII, LHCSR3, and CP43 proteins in HL cells was determined using a specific antibody (D; chlorophyll loading in each lane is reported on the top of the figure). LHCBM4/6/8, LHCBM1 (the intermediate band recognized by α-LHCII antibody), and LHCSR level per PSII (normalized to CP43 content) are reported (E, F, G). The mean value of three independent measurements (*n*=3) and the respective SDs are shown.

The effect of *LHCBM4/6/8* gene silencing on the stability of PSII was monitored *in vivo* by measuring the maximum quantum efficiency of PSII, *F*_v_/*F*_m_, by pulse-amplitude fluorimetry. *F*_v_/*F*_m_ values were found to be similar in wild-type and knock-down strains, scoring between 0.6 and 0.7 in all genotypes (Table 2). Similar values were obtained when cells were grown under high irradiance (400 µmol m^−2^ s^−1^), implying that PSII was functional even at high excitation pressure (Table 2). In order to evaluate the role of LHCBM4/6/8 in light harvesting, PSI and PSII functional antenna size was measured in dark-adapted wild-type and knock-down strains as previously described (Bonente *et al.*, 2012). In the case of PSI (Fig. 6A), functional antenna size was measured from the kinetics of P700 oxidation in thylakoid membranes treated with DCMU and methyl viologen. In particular, PSI antenna size was estimated as the reciprocal of the exponential lifetime (1/τ) obtained by fitting the oxidation kinetics with the exponential function (Bonente *et al.*, 2012). The kinetics were similar in all genotypes analyzed, and the (1/τ) values obtained for silencing were not statistically significant compared with the wild type. The antenna size of PSII was measured from the kinetics of Chl *a* fluorescence emission in DCMU-treated cells: fluorescence kinetics were fitted with exponential function by which the times required to reach two-thirds of the maximum fluorescence emission (τ_2/3_) were calculated. The reciprocal of the τ_2/3_ values was then used to estimate the PSII antenna size (Fig. 6D) as previously reported (Cardol *et al.*, 2008). No significant difference was detected for 1/τ_2/3_ values in silencing strains compared with the wild type, suggesting that the LHCII trimers destabilized upon *LHBCM4/6/8* silencing are not essential for light harvesting function, consistent with the hypothesis that it belongs to the ‘extra’ LHCII pool free in the thylakoid membranes (Drop *et al.*, 2014*a*). We then proceeded to verify the effects on regulative processes associated with the antenna system. In particular, we investigated if depletion of LHCBM4/6/8 affected the process of state 1–state 2 transitions, namely the migration of LHCII from PSII to PSI. The amplitude of state transitions was evaluated by measuring the differences in fluorescence emission upon pausing cells in either state 1 or state 2 at room temperature (Fleischmann *et al.*, 1999; Wollman, 2001) or at 77K (Allorent *et al.*, 2013). Room temperature florescence emission from whole cells essentially comes from PSII, as the fluorescence quantum yield of PSI is extremely low (Borisov and Il’ina, 1973): changes in maximum fluorescence emission at room temperature upon induction of state 1 to state 2 transition are reported in Fig. 7A, showing a similar amplitude for wild-type and knock-down strains. In order to investigate the effect of state transition on PSI, fluorescence emission spectra from whole cells in either state 1 or state 2 were also measured at 77K (Fig. 7B): 77K fluorescence emission spectra were characterized by two major peaks at 682 nm and 715 nm (Supplementary Fig. S7), related to PSII and PSI emissions, respectively. When cells were induced to state 2, PSI fluorescence emission increased more, upon normalization to PSII fluorescence, in the wild type compared with knock-down strains, suggesting LHCBM4/6/8 is part of the mobile LHCII pool transferred upon state transitions, increasing the antenna size of PSI (Drop *et al.*, 2014*b*; Le Quiniou *et al.*, 2015). In particular, since the PSII fluorescence emission measured at room temperature decreased similarly in wild-type and knock-down strains upon transition to state 2, the LHCBM4/6/8 subunits involved in state transitions are probably those located free in the membrane. The role of LHCBM4/6/8 in excess energy dissipation was evaluated by measuring the NPQ. Since in *C. reinhardtii* NPQ is fully activated upon acclimation to high light (Peers *et al.*, 2009; Bonente *et al.*, 2012; Allorent *et al.*, 2013), these measurements were performed upon acclimation to 400 µmol m^−2^ s^−1^ light. In these conditions, the number of LHCBM4/6/8 subunits per PSII in the wild type was comparable with that of cells grown in control light, and the decrease of LHCBM4/6/8 in knock-down strains was maintained (Fig. 8C, E). Knock-down strains acclimated to high light were characterized by a reduced NPQ activity (Fig. 8D), which was more evident in strain L_468. This result suggests a possible role for LHCBM4/6/8 in NPQ activity.

### Roles of LHCBM4/6/8 in stabilizing LHCSR3

Differences in NPQ induction could be related to a different accumulation of LHCSR1–LHCSR3, since LHCSR proteins are essential for triggering NPQ in *C. reinhardtii* ([Bibr CIT0063][Bibr CIT0011], 2012). The accumulation of LHCSR proteins was thus investigated by immunoblot analysis in samples grown in high light, yielding a slightly lower level of LHCSR3 in all knock-down strains as compared with the wild type (Fig. 8C, E), suggesting a possible role for LHCBM4/6/8 in stabilizing LHCSR3 in thylakoid membranes. LHCBM1 has been suggested to be partner for LHCSR3 since its depletion in the *npq5* mutant caused a strong reduction in NPQ activity (Elrad *et al.*, 2002; Peers *et al.*, 2009; Bonente *et al.*, 2011). The LHCBM1 level was measured by immunoblot analysis in the knock-down samples grown in high light, showing no significant difference as compared with the wild type (Fig. 8C, E). As reported in Supplementary Fig. S8, a positive linear correlation was found between the LHCBM4/6/8 accumulation and NPQ activation, but only for NPQ values >0.6. In contrast, no such linear correlation was found between NPQ induction and LHCBM1 or LHCSR accumulation, suggesting that the NPQ phenotype observed in silenced strains was specifically related to LHCBM4/6/8 subunits. The potential role of LHCBM4/6/8 as a binding site for LHCSR protein was then investigated by 2D electrophoresis on CN–SDS–PAGE of solubilized thylakoids from samples grown in high light conditions (Supplementary Fig. S9) coupled with immunoblot analysis using antibodies directed to PSI and PSII core subunits (PsaA and CP43) and to antenna components (LHCBM4/68 and LHCSR). In all clones the LHCSR protein was detected with mobility corresponding to that of monomeric LHC proteins or higher. The appearance of LHCSR signals at high apparent molecular weight in CN–PAGE, although weak, suggests formation of oligomers and or interactions with other thylakoid components (Bonente *et al.*, 2011; Tokutsu and Minagawa, 2013; Xue *et al.*, 2015). It should be noted that the LHCSR-specific reaction was very weak at the mobility corresponding to LHCII trimers, inconsistent with the presence of LHC heterotrimers including LHCSR3. We cannot exclude, however, the formation of LHCSR3 homodimers or heterodimers with other LHC subunits, which might then interact with PSI and/or PSII supercomplexes. The distribution patterns of LHCSR3 and LHCBM4/6/8 in CN–PAGE were different in each strain investigated. Moreover, LHCBM4/6/8 strong reduction observed in the L_468 strain did not significantly influence the LHCSR3 distribution compared with the wild type; these results suggest that LHCBM4/6/8 and LHCSR1/3 do not form stable interactions with each other.

### Roles of LHCBM4/6/8 in stress defense

**Fig. 9.**
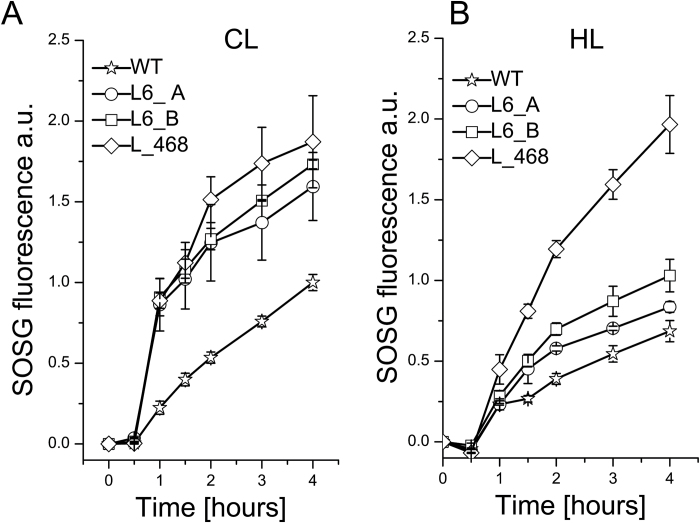
Singlet oxygen (^1^O_2_) production in knock-down strains. Singlet oxygen production was measured in cells grown in control light (A) or high light (B) conditions upon exposure to red light at 840 µmol photons m^−2^ s^−1^ following the increase of the 530 nm fluorescence of the specific probe Singlet Oxygen Sensor Green (SOSG). SDs are reported for each sample (*n*=3).

In order to investigate further the role of LHCBM4/6/8 in stress defense, the production of singlet oxygen (^1^O_2_) was measured (Fig. 9). ^1^O_2_ is produced from the reaction of molecular oxygen with chlorophyll triplet excited states and accumulates when the rate of excitation energy quenching is exceeded. ^1^O_2_ production was measured by using a specific probe, SOSG, which increases its fluorescence at 530 nm proportionally to the accumulation of ^1^O_2_ (Flors *et al.*, 2006). Cells acclimated in low light (60 µmol photons m^−2^ s^−1^) and in high light (400 µmol photons m^−2^ s^−1^) conditions were incubated in the presence of SOSG and excited by a red (680 nm) light at 840 µmol photons m^−2^ s^−1^. ^1^O_2_ production was higher in the strains acclimated in low light than in high light, suggesting that the growth in high light activates several photoprotective mechanisms decreasing photo-oxidative stress, in agreement with previous reports (Baroli *et al.*, 2003; Bonente *et al.*, 2012; Allorent *et al.*, 2013). Increased ^1^O_2_ production was observed in knock-down strains acclimated to both low and high light as compared with the wild type. In particular, strain L_468 showed the highest ^1^O_2_ production both in control light and in high light. These observations suggest a role for LHCBM4/6/8 in the mechanism of acclimation to high light conditions and photoprotection. One of the processes activated upon high light exposure is the xanthophyll cycle, during which violaxanthin is converted to zeaxanthin and anteraxanthin. The xanthophyll cycle activity can be estimated from the de-epoxidation index (DI), calculated as: (zeaxanthin+0.5×anteraxanthin)/(violaxanthin+zeaxanthin+anteraxanthin). A high DI was generally observed in high light-acclimated cells (Bonente *et al.*, 2012). Upon high light acclimation, a lower DI was observed in the silenced strains compared with the wild type (Table 2). This result suggests that the violaxanthin bound by LHCBM4/6/8 proteins can be more easily de-epoxidated to zeaxanthin as compared with the violaxanthin bound by other LHC proteins. The reduced DI observed in silenced strains in high light compared with the wild type could be related to the higher singlet oxygen production observed in these strains due to the high efficiency of zeaxanthin in scavenging ROS (Havaux and Niyogi, 1999).

## Discussion

The *LHCBM* gene family is composed of nine members, which are highly similar to each other. The functional roles of LHCBM1, LHCBM2/7, and LHCBM9 have been previously described: LHCBM1 was reported to be involved in NPQ induction, while LHCBM2/7 is involved in induction of state transitions (Elrad *et al.*, 2002; Ferrante *et al.*, 2012). The LHCBM9 subunit was found to accumulate in stress conditions only and was accompanied by an increased photoprotection activity (Nguyen *et al.*, 2008), as shown by the stabilization of both PSII supercomplexes and LHCII trimers (Grewe *et al.*, 2014). Little information was yet available for the remaining LHCBM subunits: combined silencing of LHCBM1, LHCBM2, and LHCBM3 was reported to increase light-driven hydrogen production (Oey *et al.*, 2013). Recently LHCBM1, LHCBM2/7, and LHCBM3 were demonstrated to be the major components of the heterotrimers bound to PSII supercomplexes, while LHCBM5 was suggested to be mainly located in the ‘extra’ LHCIIs which are not tightly connected to the PSII core complex (Drop *et al.*, 2014*a*). In agreement with these findings, LHCBM5 has been reported to be phosphorylated by STT7 kinase and was found in a complex with PSI upon state 2 induction (Takahashi *et al.*, 2006). It should be noted that, besides LHCBM5, also LHCBM1, LHCBM3, LHCBM4, LHCBM6, LHCBM8, and LHCBM9 can be phosphorylated by STT7, and all the different types of LHCBMs together with CP26 and CP29 were found in the PSI–LHCII supercomplex, even in non-phosphorylated form (Lemeille *et al.*, 2009; Drop *et al.*, 2014*b*).

In this work we analyzed the functional role of LHCBM4, LHCBM6, and LHCBM8 subunits, which belong to the same subfamily and share high identity (Fig. 1). The biochemical and spectroscopic features of LHCBM4 and LHCBM6 subunits were first analyzed *in vitro* and their physiological function was then studied *in vivo* by a reverse genetic approach obtaining strains silencing *LHCBM4* and *LHCBM6* (L6_A and L6_B) or the *LHCBM4/6/8* (L_468) genes together. Pigment-binding properties of LHCBM4 and LHCBM6 (Table 1) were comparable with those previously reported for other LHCBM proteins (Grewe *et al.*, 2014; Natali and Croce, 2015). An important property was their low fluorescence yield, consistently measured for both LHCBM4 and LHCBM6 as compared with LHCBM2 (Fig. 2). Since fluorescence yield is modulated by the activity of the concurrent heat dissipation channel, it can be concluded that LHCBM4 and LHCBM6 are characterized by higher quenching activity compared with LHCBM2, but comparable with LHCBM1, the LHCBM subunit with the lowest fluorescence quantum yield (Elrad *et al.*, 2002; Grewe *et al.*, 2014; Natali and Croce, 2015). The reverse genetic experiments reported here were aimed at understanding how the biochemical/biophysical properties of the individual gene products are translated into a functional role when integrated into thylakoid membranes. Analysis of selected knock-down strains showed that amiRNA silencing was effective in reducing the level of gene products *in vivo* (Fig. 4). The levels of LHCBM4/6/8 subunits were reduced on a chlorophyll basis in knock-down strains, especially in the case of L_468 (Fig. 3). Although the amiRNA silencing showed minor untargeted effect on other *LHCBM* genes, the overall stoichiometry of LHCII proteins per PSII was not significantly reduced in knock-down strains (Fig. 4). LHCBM6 accumulation has been reported to be controlled by the translation repressor NAB1, which is accumulated under CO_2_ deficiency, inducing an overall reduction in LHCII content and functional antenna size of PSII when cells are grown in the absence of CO_2_ (Berger *et al.*, 2014). The similar LHCII per PSII stoichiometry and the similar PSII antenna size observed in silencing strains in this work suggest that the translational control of NAB1 is probably not limited to LHCBM6 but involves other LHCBM subunits as well. In *C. reinhardtii*, PSII supercomplexes have been reported to have a larger capacity to bind LHCII trimers compared with higher plants, their antenna moiety in supercomplexes being constituted by at least six LHCII trimers in the C2S2M2N2 conformation, compared with the four LHCII trimers observed in *Arabidopsis thaliana* (C2S2M2) (Drop *et al.*, 2014*a*). In addition, a pool of ‘extra’ LHCII was identified in *C. reinhardtii*, constituting LHCII-only domains in the thylakoid membranes, possibly acting as a buffer for state transitions.

**Table 1.** HPLC analysis of pigment content in the recombinant and reconstituted LHCBM proteins LHCBM1, LHCBM2, LHCBM4, and LHCBM6 The numbers of each pigment are expressed in picomoles, and normalized to 14 chlorophylls (the number of chlorophylls putatively bound by one LHCII monomer).

**Refolded complexes**	**Chl**	**Chl *a/b***	**Chl/Car**	**Cars**	**Nx**	**Vx**	**Ax**	**Lut**	**Zx**
LHCBM1	14	1.41	4.1	3.4	1.5	0.28	0.03	1.54	0.034
LHCBM2	14	1.148	3.89	3.6	1.63	0.2	0.01	1.73	0.022
LHCBM4	14	1.3	3.41	4.1	2	0.24	0.012	1.81	0.026
LHCBM6	14	1.37	3.3	4.23	2.19	0.18	0.03	1.76	0.066

Chl, chlorophylls; Chl *a/b*, chlorophyll *a*/b ratio; Chl/Car, chlorophyll to carotenoid ratio, Cars, total carotenoids; Nx, neoxanthin; Vx, violaxanthin; Ax, antheraxanthin; Lut, lutein; Zx, zeaxanthin.

Chl *a/b* and Chl/Car ratios are absolute values.

SDs are in all cases <5% (*n*=3).

The results obtained by 2D CN–SDS–PAGE showed that LHCBM4/6/8 contribute to form monomeric and trimeric LHC bands or to PSII supercomplexes of different sizes. This evidence suggests that LHCBM4/6/8 can be part of -S, -M, or -N trimers. Nevertheless, their enrichment in supercomplexes was low, and most of LHBCM4/6/8 was found in the ‘free LHCII’ pool (Supplementary Fig. S6). In agreement with this finding, PSII antenna size was essentially unaffected by *LHCBM4/6/8* gene silencing. LHCII trimers free in the thylakoid membrane are suggested to be bound to PSI or forming LHCII-only domains (Nagy *et al.*, 2014; Ünlü *et al.*, 2014). When wild-type and knock-down strains were forced to undergo transition to state 2, the PSII fluorescence emission was similarly reduced in wild-type and knock-down strains, while the increase of PSI fluorescence emission, detectable at 77K, was significantly smaller in knock-down strains compared with the wild type, indicating a reduced level of LHCII–PSI interaction. On this basis, we suggest that LHCBM4/6/8 are located in substoichiometric amounts in -S, -M, or -N trimers, while the majority of these subunits are located free in the membrane, with the latter participating in state transitions (i.e. migrating to PSI upon state 2 induction). The same conclusion can be extended to the other LHCII subunits forming heterotrimes with LHCBM4/6/8.

The down-regulation of LHCBM4/6/8 protein was correlated with a decrease in the amplitude of NPQ activity (Fig. 8; Table 2; Supplementary Fig. S8). The high sequence identity of LHCBM4, LHCBM6, and LHCBM8 suggests that these proteins have similar functions, acting co-operatively, in the energy dissipative mechanisms. How LHCBM4/6/8 contribute to NPQ is not clear. One possibility is that they are docking site(s) for the interaction of the PSII antenna system with LHCSR3, which, owing to its short fluorescence lifetime upon lumen acidification, could act as the site for energy dissipation (Peers *et al.*, 2009; Bonente *et al.*, 2011; Liguori *et al.*, 2013; Tokutsu and Minagawa, 2013). Alternatively, it is possible that quenching sites are formed not only within LHCSR1/3 proteins but also in the interacting LHC subunits induced to switch to a dissipative conformation by the interaction with LHCSR proteins, in a mechanism similar to what was previously proposed for the PSBS-dependent quenching in higher plants (Bonente *et al.*, 2008). While the present data do not allow us to distinguish between these hypotheses, the interaction between LHCSR3 and other pigment proteins appears to be very weak, at least in the fractionation conditions explored here. Indeed, the LHCSR distribution was not affected in knock-down strains (Supplementary Fig. S9). Thus, it is unlikely that LHCSR3 might form stable hetero-oligomers with LHCBM4/6/8. It is, however, possible that the relative abundance of high versus low fluorescence yield LHCM subunits might serve in the fine-tuning of the antenna system during long-term acclimation consistent, with the recent results with LHCBM9 (Grewe *et al.*, 2014) and with the LHCII populations with different quenching properties detected *in vivo* (Tian *et al.*, 2015), rather than on the light-induced short-term NPQ mechanism. A role for LHCBM4/6/8 in the formation of quenched LHCII domains is also consistent with the higher level of singlet oxygen in knock-down strains compared with the wild type (Fig. 9) during growth in both control and high light conditions. The level of ROS produced upon light exposure in pigment–protein antennas depends on the level of chlorophyll singlet excited states, the conversion yield into triplets, and the ROS-scavenging activity of xanthophylls (Ballottari *et al.*, 2013; Croce *et al.*, 1999*b*; Niyogi, 1999). Certainly, the reduced capacity for NPQ is likely to contribute to ROS synthesis in excess light conditions (Ferrante *et al.*, 2012). However, differences in ROS-scavenging activity cannot be excluded, especially considering the decrease of the de-epoxidation index measured in these strains (Table 2). Indeed, zeaxanthin has been involved in quenching of singlet chlorophyll excited states (Dall’Osto *et al.*, 2005), quenching of triplet chlorophyll excited states (Dall’Osto *et al.*, 2012), and ROS scavenging (Havaux *et al.*, 2004). Interestingly, while singlet oxygen production in high light-acclimated cells was generally lower, this was not the case in the L_468 strain, whose high light-acclimated cells produced levels of singlet oxygen comparable with cells receiving control light. These results, together with the reduced LHCSR3 accumulation and reduced de-epoxidation index in the L_468 strain, suggest that the reduction in level of the LHCBM4/6/8 proteins impairs the mechanisms of acclimation to high light.

**Table 2.** F_v_/F_m_ and NPQ parameter of wild-type (WT) and knock-down strains F_v_/F_m_ values were determined by PAM fluorimetery on cells grown in control light (CL) or high light (HL). NPQ values were measured by PAM fluorimetry on HL cells.

	***F***_**v**_ **/*F***_**m**_ **(CL)**	***F***_**v**_ **/*F***_**m**_ **(HL)**	**NPQ_max**
WT	0.712 ± 0.02	0.663 ± 0.01	1.57 ± 016
L6_A	0.701 ± 0.01	0.628 ± 0.01	0.98 ± 0.04
L6_B	0.703 ± 0.02	0.663 ± 0.01	1.12 ± 0.03
L_468	0.709 ± 0.01	0.660 ± 0.01	0.54 ± 0.01

The SD dis reported in the table (*n*=5).

**Table 3.** Pigment profiling of knock-down strains grown in control light (CL) and high light (HL) Pigment amounts quantified by HPLC are normalized to 100 chlorophyll.

	**Nx**	**Vx**	**Ax**	**Lut**	**Zx**	**β-Car**	**Chl *a/b***	**Car/Chl**	**DI**	**Chl/cell**
WT CL	9.3 ± 1.2	4.2 ± 0.5	0.27 ± 0.03	7.5 ± 0.9	0.22 ± 0.03	4.6 ± 0.1	2,19 ± 0,03	0.26 ± 0.02	0.076 ± 0.002	2,7E-06 ± 6,7E-08
L6_A CL	10.2 ± 0.04	4.9 ± 0.01	0.3 ± 0.01	8.4 ± 0.03	0.34 ± 0.03	5.01 ± 0.08	2,23 ± 0,05	0.29 ± 0.002	0.089 ± 0.004	2,0E-06 ± 1,7E-07
L6_B CL	9.4 ± 1.2	4.7 ± 0.8	0.3 ± 0.06	8.4 ± 1.1	0.16 ± 0.06	5.2 ± 2.1	2,26 ± 0,02	0.28 ± 0.05	0.064 ± 0.016	2,7E-06 ± 2,8E-07
L_468 CL	8.7 ± 1.1	4.2 ± 0.5	0.27 ± 0.04	7.4 ± 0.9	0.25 ± 0.05	4.8 ± 0.1	2,36 ± 0,02	0.26 ± 0.03	0.08 ± 0.005	2,9E-06 ± 4,0E-07
WT HL	7.7 ± 2.3	4.4 ± 1.6	1.4 ± 0.5	16.1 ± 4.8	1.7 ± 0.8	2.7 ± 1.9	2,22 ± 0,00	0.35 ± 0.14	0.316 ± 0.01	1,5E-06 ± 7,7E-08
L6_A HL	7.1 ± 1.6	5.1 ± 1.4	0.9 ± 0.1	15.4 ± 2,0	0.9 ± 0.003	1.4 ± 0.001	2,31 ± 0,01	0.35 ± 0.0	0.196 ± 0.03	1,4E-06 ± 1,1E-07
L6_ B HL	6.6 ± 0.9	4.9 ± 0.8	0.74 ± 0.1	13.2 ± 1.8	0.74 ± 0.1	4.99 ± 0.2	2,31 ± 0,07	0.31 ± 0.04	0.172 ± 0.0003	1,7E-06 ± 1,7E-07
L_468 HL	8.1 ± 1.4	5.7 ± 0.8	0.7 ± 0.08	14.2 ± 2.1	0.6 ± 0.04	4.5 ± 1.5	2,39 ± 0,00	0.34 ± 0.06	0.135 ± 0.002	2,0E-06 ± 2,1E-07

Nx, neoxanthin; Vx, violaxanthin; Ax, antheraxanthin, Lut, lutein, Zx, zeaxanthin, β-Car, β-carotene, Chl *a/b*, the ratio between Chl *a* and Chl *b*; Car/Chl, the ratio between the total carotenoid and chlorophyll content; DI, de-epoxidation index: DI=Zx+(0.5×Ax)/(Vx+Ax+Zx).

The SD is reported in the table (*n*=3).

We conclude that LHCBM4, LHCBM6, and LHCBM8, rather than having an essential function in photon capture, are likely to be involved in photoprotective mechanisms with a specific function within a pool of LHCII proteins free or very loosely connected to the PSII supercomplex. Beside their interest for the understanding of basic properties of light-harvesting systems, these results will also be instrumental in designing domesticated strains of unicellular algae for optimal growth in photobioreactors by modulating the accumulation of specific members of the antenna system in order to improve either light harvesting, the photoprotection response, or both.

## Supplementary Material

Supplementary Data

## Abbreviations:

amiRNA: artificial microRNA
CN: Clear Native
DCMU: [3-(3,4-dichlorophenyl)-1,1-dimethylurea]
DI: de-epoxidation index
LHC: light-harvesting complex
NPQ: non-photochemical quenching
^1^O_2_: singlet oxygen
ROS: reactive oxygen species.

## References

[CIT0001] Ahn TK Avenson TJ Ballottari M Cheng YC Niyogi KK Bassi R Fleming GR 2008 Architecture of a charge-transfer state regulating light harvesting in a plant antenna protein. Science 320, 794–797. 18467588 10.1126/science.1154800

[CIT0002] Allen JF Pfannschmidt T 2000 Balancing the two photosystems: photosynthetic electron transfer governs transcription of reaction centre genes in chloroplasts. Philosophical Transactions of the Royal Society B: Biological Sciences 355, 1351–1359. 10.1098/rstb.2000.0697 PMC1692884 11127990

[CIT0003] Allorent G Tokutsu R Roach T 2013 A dual strategy to cope with high light in Chlamydomonas reinhardtii. The Plant Cell 25, 545–557. 23424243 10.1105/tpc.112.108274 PMC3608777

[CIT0004] Ballottari M Girardon J Dall’osto L Bassi R 2012 Evolution and functional properties of photosystem II light harvesting complexes in eukaryotes. Biochimica et Biophysica Acta 1817, 143–157. 21704018 10.1016/j.bbabio.2011.06.005

[CIT0005] Ballottari M Mozzo M Croce R Morosinotto T Bassi R 2009 Occupancy and functional architecture of the pigment binding sites of photosystem II antenna complex Lhcb5. Journal of Biological Chemistry 284, 8103–8113. 19129188 10.1074/jbc.M808326200 PMC2658104

[CIT0006] Ballottari M Mozzo M Girardon J Hienerwadel R Bassi R 2013 Chlorophyll triplet quenching and photoprotection in the higher plant monomeric antenna protein Lhcb5. Journal of Physical Chemistry B 117, 11337–11348. 10.1021/jp402977y 23786371

[CIT0007] Baroli I Do AD Yamane T Niyogi KK 2003 Zeaxanthin accumulation in the absence of a functional xanthophyll cycle protects Chlamydomonas reinhardtii from photooxidative stress. The Plant Cell 15, 992–1008. 12671093 10.1105/tpc.010405 PMC152344

[CIT0008] Benson SL Maheswaran P Ware MA Hunter CN Horton P Jansson S Ruban AV Johnson MP 2015 An intact light harvesting complex I antenna system is required for complete state transitions in Arabidopsis. Nature Plants 1, 15176. 27251716 10.1038/nplants.2015.176

[CIT0009] Berger H Blifernez-Klassen O Ballottari M Bassi R Wobbe L Kruse O 2014 Integration of carbon assimilation modes with photosynthetic light capture in the green alga Chlamydomonas reinhardtii. Molecular Plant 7, 1545–1559. 25038233 10.1093/mp/ssu083

[CIT0010] Betterle N Ballottari M Baginsky S Bassi R 2015 High light-dependent phosphorylation of photosystem II inner antenna CP29 in monocots is STN7 independent and enhances nonphotochemical quenching. Plant Physiology 167, 457–471. 25501945 10.1104/pp.114.252379 PMC4326754

[CIT0011] Bonente G Ballottari M Truong TB Morosinotto T Ahn TK Fleming GR Niyogi KK Bassi R 2011 Analysis of LhcSR3, a protein essential for feedback de-excitation in the green alga Chlamydomonas reinhardtii. PloS Biology 9, e1000577. 21267060 10.1371/journal.pbio.1000577 PMC3022525

[CIT0012] Bonente G Howes BD Caffarri S Smulevich G Bassi R 2008 Interactions between the photosystem II subunit PsbS and xanthophylls studied in vivo and in vitro. Journal of Biological Chemistry 283, 8434–8445. 18070876 10.1074/jbc.M708291200 PMC2417184

[CIT0013] Bonente G Pippa S Castellano S Bassi R Ballottari M 2012 Acclimation of Chlamydomonas reinhardtii to different growth irradiances. Journal of Biological Chemistry 287, 5833–5847. 22205699 10.1074/jbc.M111.304279 PMC3285353

[CIT0014] Borisov AY Il’ina MD 1973 The fluorescence lifetime and energy migration mechanism in photosystem I of plants. Biochimica et Biophysica Acta 305, 364–371. 4741135 10.1016/0005-2728(73)90182-5

[CIT0015] Caffarri S Croce R Breton J Bassi R 2001 The major antenna complex of photosystem II has a xanthophyll binding site not involved in light harvesting. Journal of Biological Chemistry 276, 35924–35933. 11454869 10.1074/jbc.M105199200

[CIT0016] Caffarri S Croce R Cattivelli L Bassi R 2004 A look within LHCII: differential analysis of the Lhcb1–3 complexes building the major trimeric antenna complex of higher-plant photosynthesis. Biochemistry 43, 9467–9476. 15260489 10.1021/bi036265i

[CIT0017] Caffarri S Passarini F Bassi R Croce R 2007 A specific binding site for neoxanthin in the monomeric antenna proteins CP26 and CP29 of photosystem II. FEBS Letters 581, 4704–4710. 17850797 10.1016/j.febslet.2007.08.066

[CIT0018] Cardol P Bailleul B Rappaport F 2008 An original adaptation of photosynthesis in the marine green alga Ostreococcus. Proceedings of the National Academy of Sciences, USA 105, 7881–7886. 10.1073/pnas.0802762105 PMC2409423 18511560

[CIT0019] Croce R Morosinotto T Castelletti S Breton J Bassi R 2002 The Lhca antenna complexes of higher plants photosystem I. Biochimica et Biophysica Acta 1556, 29–40. 12351216 10.1016/s0005-2728(02)00304-3

[CIT0020] Croce R Remelli R Varotto C Breton J Bassi R 1999 *a* The neoxanthin binding site of the major light harvesting complex (LHCII) from higher plants. FEBS Letters 456, 1–6. 10452518 10.1016/s0014-5793(99)00907-2

[CIT0021] Croce R Weiss S Bassi R 1999 *b* Carotenoid-binding sites of the major light-harvesting complex II of higher plants. Journal of Biological Chemistry 274, 29613–29623. 10514429 10.1074/jbc.274.42.29613

[CIT0022] Dall’Osto L Caffarri S Bassi R 2005 A mechanism of nonphotochemical energy dissipation, independent from PsbS, revealed by a conformational change in the antenna protein CP26. The Plant Cell 17, 1217–1232. 15749754 10.1105/tpc.104.030601 PMC1087998

[CIT0023] Dall’Osto L Cazzaniga S Havaux M Bassi R 2010 Enhanced photoprotection by protein-bound vs free xanthophyll pools: a comparative analysis of chlorophyll b and xanthophyll biosynthesis mutants. Molecular Plant 3, 576–593. 20100799 10.1093/mp/ssp117

[CIT0024] Dall’Osto L Cazzaniga S North H Marion-Poll A Bassi R 2007 The Arabidopsis aba4-1 mutant reveals a specific function for neoxanthin in protection against photooxidative stress. The Plant Cell 19, 1048–1064. 17351115 10.1105/tpc.106.049114 PMC1867355

[CIT0025] Dall’Osto L Holt NE Kaligotla S Fuciman M Cazzaniga S Carbonera D Frank HA Alric J Bassi R 2012 Zeaxanthin protects plant photosynthesis by modulating chlorophyll triplet yield in specific light-harvesting antenna subunits. Journal of Biological Chemistry 287, 41820–41834. 23066020 10.1074/jbc.M112.405498 PMC3516730

[CIT0026] Dall’Osto L Lico C Alric J Giuliano G Havaux M Bassi R 2006 Lutein is needed for efficient chlorophyll triplet quenching in the major LHCII antenna complex of higher plants and effective photoprotection in vivo under strong light. BMC Plant Biology 6, 32. 17192177 10.1186/1471-2229-6-32 PMC1769499

[CIT0027] Dall’Osto L Piques M Ronzani M Molesini B Alboresi A Cazzaniga S Bassi R 2013 The Arabidopsis nox mutant lacking carotene hydroxylase activity reveals a critical role for xanthophylls in photosystem I biogenesis. The Plant Cell 25, 591–608. 23396829 10.1105/tpc.112.108621 PMC3608780

[CIT0028] de Bianchi S Betterle N Kouril R Cazzaniga S Boekema E Bassi R Dall’Osto L 2011 Arabidopsis mutants deleted in the light-harvesting protein Lhcb4 have a disrupted photosystem II macrostructure and are defective in photoprotection. The Plant Cell 23, 2659–2679. 21803939 10.1105/tpc.111.087320 PMC3226214

[CIT0029] Depège N Bellafiore S Rochaix JD 2003 Role of chloroplast protein kinase Stt7 in LHCII phosphorylation and state transition in Chlamydomonas. Science 299, 1572–1575. 12624266 10.1126/science.1081397

[CIT0030] Drop B Webber-Birungi M Yadav SK Filipowicz-Szymanska A Fusetti F Boekema EJ Croce R 2014 *a* Light-harvesting complex II (LHCII) and its supramolecular organization in Chlamydomonas reinhardtii. Biochimica et Biophysica Acta 1837, 63–72. 23933017 10.1016/j.bbabio.2013.07.012

[CIT0031] Drop B Yadav KNS Boekema EJ Croce R 2014 *b* Consequences of state transitions on the structural and functional organization of photosystem I in the green alga Chlamydomonas reinhardtii. The Plant Journal 78, 181–191. 24506306 10.1111/tpj.12459

[CIT0032] Elrad D Niyogi KK Grossman AR 2002 A major light-harvesting polypeptide of photosystem II functions in thermal dissipation. The Plant Cell 14, 1801–1816. 12172023 10.1105/tpc.002154 PMC151466

[CIT0033] Ferrante P Ballottari M Bonente G Giuliano G Bassi R 2012 LHCBM1 and LHCBM2/7 polypeptides, components of major LHCII complex, have distinct functional roles in photosynthetic antenna system of Chlamydomonas reinhardtii. Journal of Biological Chemistry 287, 16276–16288. 22431727 10.1074/jbc.M111.316729 PMC3351333

[CIT0034] Finazzi G Rappaport F Furia A Fleischmann M Rochaix JD Zito F Forti G 2002 Involvement of state transitions in the switch between linear and cyclic electron flow in Chlamydomonas reinhardtii. EMBO Reports 3, 280–285. 11850400 10.1093/embo-reports/kvf047 PMC1084013

[CIT0035] Fleischmann MM Ravanel S Delosme R Olive J Zito F Wollman FA Rochaix JD 1999 Isolation and characterization of photoautotrophic mutants of Chlamydomonas reinhardtii deficient in state transition. Journal of Biological Chemistry 274, 30987–30994. 10521495 10.1074/jbc.274.43.30987

[CIT0036] Flors C Fryer MJ Waring J Reeder B Bechtold U Mullineaux PM Nonell S Wilson MT Baker NR 2006 Imaging the production of singlet oxygen in vivo using a new fluorescent sensor, Singlet Oxygen Sensor Green. Journal of Experimental Botany 57, 1725–1734. 16595576 10.1093/jxb/erj181

[CIT0037] Galka P Santabarbara S Khuong TT Degand H Morsomme P Jennings RC Boekema EJ Caffarri S 2012 Functional analyses of the plant photosystem–light-harvesting complex II supercomplex reveal that light-harvesting complex II loosely bound to photosystem II is a very efficient antenna for photosystem I in state II. The Plant Cell 24, 2963–2978. 22822202 10.1105/tpc.112.100339 PMC3426126

[CIT0038] Giuffra E Cugini D Croce R Bassi R 1996 Reconstitution and pigment-binding properties of recombinant CP29. European Journal of Biochemistry 238, 112–120. 8665927 10.1111/j.1432-1033.1996.0112q.x

[CIT0039] Grewe S Ballottari M Alcocer M D’Andrea C Blifernez-Klassen O Hankamer B Mussgnug JH Bassi R Kruse O 2014 Light-harvesting complex protein LHCBM9 is critical for photosystem II activity and hydrogen production in Chlamydomonas reinhardtii. The Plant Cell 26, 1598–1611. 24706511 10.1105/tpc.114.124198 PMC4036574

[CIT0040] Havaux M Dall’Osto L Bassi R 2007 Zeaxanthin has enhanced antioxidant capacity with respect to all other xanthophylls in Arabidopsis leaves and functions independent of binding to PSII antennae. Plant Physiology 145, 1506–1520. 17932304 10.1104/pp.107.108480 PMC2151694

[CIT0041] Havaux M Dall’Osto L Cuiné S Giuliano G Bassi R 2004 The effect of zeaxanthin as the only xanthophyll on the structure and function of the photosynthetic apparatus in Arabidopsis thaliana. Journal of Biological Chemistry 279, 13878–13888. 14722117 10.1074/jbc.M311154200

[CIT0042] Havaux M Niyogi KK 1999 The violaxanthin cycle protects plants from photooxidative damage by more than one mechanism. Proceedings of the National Academy of Sciences, USA 96, 8762–8767. 10.1073/pnas.96.15.8762 PMC17590 10411949

[CIT0043] Havaux M Tardy F 1997 Thermostability and photostability of photosystem II in leaves of the chlorina-f2 barley mutant deficient in light-harvesting chlorophyll a/b protein complexes. Plant Physiology 113, 913–923. 12223653 10.1104/pp.113.3.913 PMC158211

[CIT0044] Hobe S Förster R Klingler J Paulsen H 1995 N-proximal sequence motif in light-harvesting chlorophyll a/b-binding protein is essential for the trimerization of light-harvesting chlorophyll a/b complex. Biochemistry 34, 10224–10228. 7640277 10.1021/bi00032a016

[CIT0045] Kindle KL 1990 High-frequency nuclear transformation of Chlamydomonas reinhardtii. Proceedings of the National Academy of Sciences, USA 87, 1228–1232. 10.1073/pnas.87.3.1228 PMC53444 2105499

[CIT0046] Kühlbrandt W Wang DN 1991 Three-dimensional structure of plant light-harvesting complex determined by electron crystallography. Nature 350, 130–134. 2005962 10.1038/350130a0

[CIT0047] Kühlbrandt W Wang DN Fujiyoshi Y 1994 Atomic model of plant light-harvesting complex by electron crystallography. Nature 367, 614–621. 8107845 10.1038/367614a0

[CIT0048] Lagarde D Beuf L Vermaas W 2000 Increased production of zeaxanthin and other pigments by application of genetic engineering techniques to Synechocystis sp. strain PCC 6803. Applied and Environmental Microbiology 66, 64–72. 10618204 10.1128/aem.66.1.64-72.2000 PMC91786

[CIT0049] Le Quiniou C van Oort B Drop B van Stokkum IH Croce R 2015 The high efficiency of photosystem I in the green alga Chlamydomonas reinhardtii is maintained after the antenna size is substantially increased by the association of light-harvesting complexes II. Journal of Biological Chemistry 290, 30587–30595. 26504081 10.1074/jbc.M115.687970 PMC4683278

[CIT0050] Lemeille S Willig A Depège-Fargeix N Delessert C Bassi R Rochaix JD 2009 Analysis of the chloroplast protein kinase Stt7 during state transitions. PLoS Biology 7, e45. 19260761 10.1371/journal.pbio.1000045 PMC2650728

[CIT0051] Li Z Ahn TK Avenson TJ Ballottari M Cruz JA Kramer DM Bassi R Fleming GR Keasling JD Niyogi KK 2009 Lutein accumulation in the absence of zeaxanthin restores nonphotochemical quenching in the Arabidopsis thaliana npq1 mutant. The Plant Cell 21, 1798–1812. 19549928 10.1105/tpc.109.066571 PMC2714924

[CIT0052] Liguori N Roy LM Opacic M Durand G Croce R 2013 Regulation of light harvesting in the green alga Chlamydomonas reinhardtii: the C-terminus of LHCSR is the knob of a dimmer switch. Journal of the American Chemical Society 135, 18339–18342. 24261574 10.1021/ja4107463

[CIT0053] Liu Z Yan H Wang K Kuang T Zhang J Gui L An X Chang W 2004 Crystal structure of spinach major light-harvesting complex at 2.72 A resolution. Nature 428, 287–292. 15029188 10.1038/nature02373

[CIT0054] Merchant SS Prochnik SE Vallon O 2007 The Chlamydomonas genome reveals the evolution of key animal and plant functions. Science 318, 245–250. 17932292 10.1126/science.1143609 PMC2875087

[CIT0055] Molnar A Bassett A Thuenemann E Schwach F Karkare S Ossowski S Weigel D Baulcombe D 2009 Highly specific gene silencing by artificial microRNAs in the unicellular alga Chlamydomonas reinhardtii. The Plant Journal 58, 165–174. 19054357 10.1111/j.1365-313X.2008.03767.x

[CIT0056] Nagy G Ünnep R Zsiros O 2014 Chloroplast remodeling during state transitions in Chlamydomonas reinhardtii as revealed by noninvasive techniques in vivo. Proceedings of the National Academy of Sciences, USA 111, 5042–5047. 10.1073/pnas.1322494111 PMC3977285 24639515

[CIT0057] Natali A Croce R 2015 Characterization of the major light-harvesting complexes (LHCBM) of the green alga Chlamydomonas reinhardtii. PLoS One 10, e0119211. 25723534 10.1371/journal.pone.0119211 PMC4344250

[CIT0058] Nawrocki WJ Santabarbara S Mosebach L Wollman FA Rappaport F 2016 State transitions redistribute rather than dissipate energy between the two photosystems in Chlamydomonas. Nature Plants 2, 16031. 27249564 10.1038/nplants.2016.31

[CIT0059] Nguyen AV Thomas-Hall SR Malnoë A Timmins M Mussgnug JH Rupprecht J Kruse O Hankamer B Schenk PM 2008 Transcriptome for photobiological hydrogen production induced by sulfur deprivation in the green alga Chlamydomonas reinhardtii. Eukaryotic Cell 7, 1965–1979. 18708561 10.1128/EC.00418-07 PMC2583537

[CIT0060] Niyogi KK 1999 Photoprotection revisited: genetic and molecular approaches. Annual Review of Plant Physiology and Plant Molecular Biology 50, 333–359. 10.1146/annurev.arplant.50.1.333 15012213

[CIT0061] Oey M Ross IL Stephens E Steinbeck J Wolf J Radzun KA Kügler J Ringsmuth AK Kruse O Hankamer B 2013 RNAi knock-down of LHCBM1, 2 and 3 increases photosynthetic H2 production efficiency of the green alga Chlamydomonas reinhardtii. PLoS One 8, e61375. 23613840 10.1371/journal.pone.0061375 PMC3628864

[CIT0062] Pan X Li M Wan T Wang L Jia C Hou Z Zhao X Zhang J Chang W 2011 Structural insights into energy regulation of light-harvesting complex CP29 from spinach. Nature Structural and Molecular Biology 18, 309–315. 10.1038/nsmb.2008 21297637

[CIT0063] Peers G Truong TB Ostendorf E Busch A Elrad D Grossman AR Hippler M Niyogi KK 2009 An ancient light-harvesting protein is critical for the regulation of algal photosynthesis. Nature 462, 518–521. 19940928 10.1038/nature08587

[CIT0064] Ruban AV Berera R Ilioaia C van Stokkum IH Kennis JT Pascal AA van Amerongen H Robert B Horton P van Grondelle R 2007 Identification of a mechanism of photoprotective energy dissipation in higher plants. Nature 450, 575–578. 18033302 10.1038/nature06262

[CIT0065] Schägger H von Jagow G 1987 Tricine–sodium dodecyl sulfate–polyacrylamide gel electrophoresis for the separation of proteins in the range from 1 to 100 kDa. Analytical Biochemistry 166, 368–379. 2449095 10.1016/0003-2697(87)90587-2

[CIT0066] Standfuss J Terwisscha van Scheltinga AC Lamborghini M Kühlbrandt W 2005 Mechanisms of photoprotection and nonphotochemical quenching in pea light-harvesting complex at 2.5 A resolution. EMBO Journal 24, 919–928. 15719016 10.1038/sj.emboj.7600585 PMC554132

[CIT0067] Takahashi H Iwai M Takahashi Y Minagawa J 2006 Identification of the mobile light-harvesting complex II polypeptides for state transitions in Chlamydomonas reinhardtii. Proceedings of the National Academy of Sciences, USA 103, 477–482. 10.1073/pnas.0509952103 PMC1326185 16407170

[CIT0068] Tian L Dinc E Croce R 2015 LHCII populations in different quenching states are present in the thylakoid membranes in a ratio that depends on the light conditions. Journal of Physical Chemistry Letters 6, 2339–2344. 26266614 10.1021/acs.jpclett.5b00944

[CIT0069] Tokutsu R Minagawa J 2013 Energy-dissipative supercomplex of photosystem II associated with LHCSR3 in Chlamydomonas reinhardtii. Proceedings of the National Academy of Sciences, USA 110, 10016–10021. 10.1073/pnas.1222606110 PMC3683755 23716695

[CIT0070] Turkina MV Kargul J Blanco-Rivero A Villarejo A Barber J Vener AV 2006 Environmentally modulated phosphoproteome of photosynthetic membranes in the green alga Chlamydomonas reinhardtii. Molecular and Cellular Proteomics 5, 1412–1425. 16670252 10.1074/mcp.M600066-MCP200

[CIT0071] Ünlü C Drop B Croce R van Amerongen H 2014 State transitions in Chlamydomonas reinhardtii strongly modulate the functional size of photosystem II but not of photosystem I. Proceedings of the National Academy of Sciences, USA 111, 3460–3465. 10.1073/pnas.1319164111 PMC3948275 24550508

[CIT0072] Wollman FA 2001 State transitions reveal the dynamics and flexibility of the photosynthetic apparatus. EMBO Journal 20, 3623–3630. 11447103 10.1093/emboj/20.14.3623 PMC125556

[CIT0073] Xue H Tokutsu R Bergner SV Scholz M Minagawa J Hippler M 2015 PHOTOSYSTEM II SUBUNIT R is required for efficient binding of LIGHT-HARVESTING COMPLEX STRESS-RELATED PROTEIN3 to photosystem II–light-harvesting supercomplexes in Chlamydomonas reinhardtii. Plant Physiology 167, 1566–1578. 25699588 10.1104/pp.15.00094 PMC4378180

[CIT0074] Zhao T Wang W Bai X Qi Y 2009 Gene silencing by artificial microRNAs in Chlamydomonas. The Plant Journal 58, 157–164. 19054364 10.1111/j.1365-313X.2008.03758.x

