# Supplementary material for: The function of LHCBM4/6/8 antenna proteins in Chlamydomonas reinhardtii
Source: J Exp Bot. 2016 Dec 22;68(3):627–41. doi: 10.1093/jxb/erw462 (PMC5441897; doi:10.1093/jxb/erw462)

SUPPLEMENTARY DATA

Table S1: Nucleotide sequence of the primers used for RT-PCR analysis

Primers used for RT-PCR analysis on *LHCBM1-9, RBCS2 (RIBULOSE-1,5-BISPHOSPHATE CARBOXYLASE SMALL-SUBUNIT)* and *CBLP (G-PROTEIN BETA SUBUNIT-LIKE POLYPEPTIDE*) genes.

**Gene**	**Forward sequence (5' -3')**	**Reverse sequence (5'-3')**
LHCBM1	TGAGCGGTTTATTTGGGTCG	GGACCTTGCCTGCTGCAC
LHCBM2	TCGAGGCCTTCTTGACTTGAGT	CCACTTGATTCACCGGCAGT
LHCBM3	GCATTTGTTGCGTCTTTTTG	AAACGCTGCGGTTTAAAAAT
LHCBM4	TGCAGGCTTTTGTTTGCTATTG	CACACGCAACATTCGAGTCAGT
LHCBM5	GCTGATGGCAAATTATTTGGGT	GGAGATGGAAAGAAAACGCG
LHCBM6	GCAAAGGATGCCCTTGTAGT	GGAATGGGCTCTTCCCTAGT
LHCBM7	ATGTACTGGCGTGATTGAGC	AATCGCAAACCAACATACCA
LHCBM8	GCCTACGAGGATGCTGAGGAT	CACCCAGCGTTAGCCACTAGC
LHCBM9	AGGCCTTCTGGATGTACCAC	ATGGTTCTGGACACAACTGC
CBLP	CGTGGCTTTCTCGGTGGA	CGCCAATGGTGTACTTGCACT
RBCS2	CCTGCCTGGAGTTCGCTG	CCAGTAGCGGTTGTCGTAGTACAG

Table S2: Nucleotide sequence of the amiRNAs used and position on *LHCBM4, LHCBM6* and *LHCBM8* mRNAs.

Two different amiRNAs (LHCBM6A and LHCBM6B) were designed for silencing *LHCBM6* gene while four different amiRNAs were designed for the simultaneous silencing of *LHCBM4*, *LHCBM6* and *LHCBM8* genes but just one (indicated in the Table) was effective in silencing the three selected genes. Hybridization energy between the amiRNA and its target is expressed as kcal/mole. The position of the mismatches is indicated in brackets and is relative to the reverse complement sequence of the amiRNA starting from the 5' nucleotide. The schematic position of the amiRNAs on the *LHCBM* mRNAs is shown in Figure S3.

**Target**	**amiRNA**	**amiRNA sequence**	**Position on mRNA**	**Hybridization energy**	**Mismatches**
LHCBM6	LHCBM6A	TTTGGAATGGGCTCTCCCCTA	3'UTR (1516-1536)	-41,31	1 (6)
LHCBM6	LHCBM6B	TAAGTGACCCAGGACAGGCAT	5'UTR (324-344)	-40,99	2 (7 and 21)
LHCBM4+6+8	LHCBM4+6+8	TAACTCAACGCCAGAGGTCTT	CDS (117-137 for *LHCBM4*; 543-563 for *LHCBM6*; 127-147 for *LHCBM8*)	-38.71	2 (5 and 21)

Figure S1: Fluorescence emission spectra of refolded recombinant LHCBM proteins. Pigments connectivity on recombinant proteins refolded *in vitro* was evaluated by measuring the fluorescence emission spectra upon excitation of chlorophyll a (440nm), chlorophyll b (475 nm) and carotenoids (500 nm). Panel A: LHCBM1. Panel B: LHCBM2. Panel C: LHCBM4. Panel D: LHCBM6.

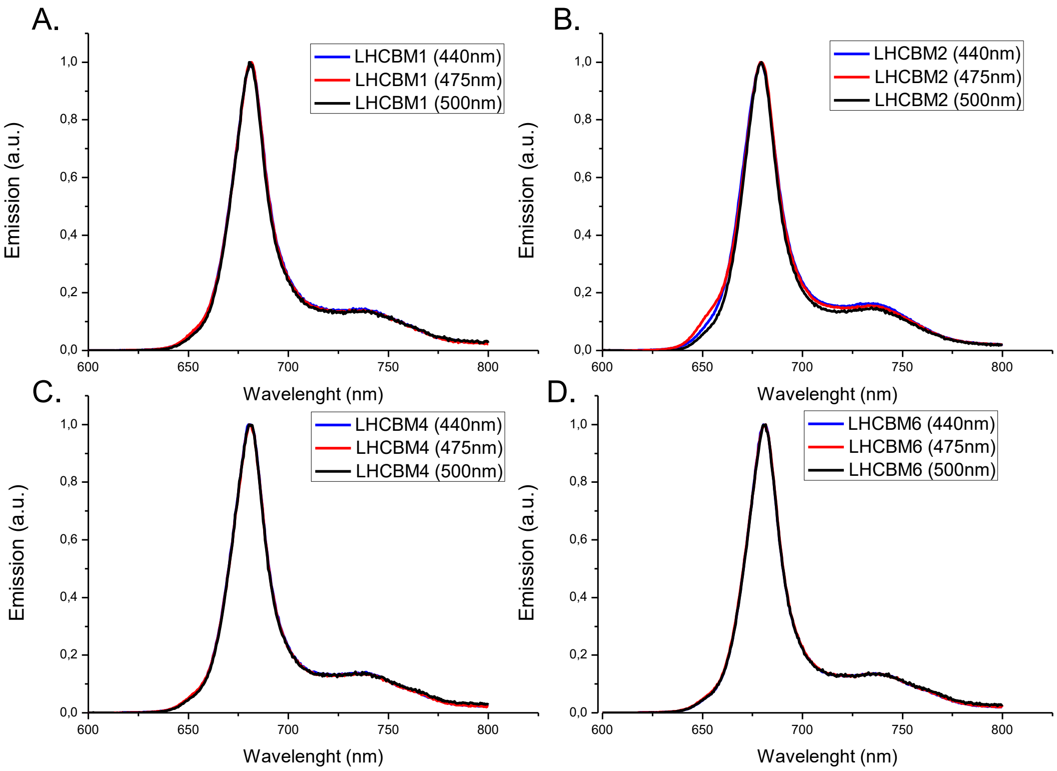

Figure S2. Evaluation of α-LHCII and α-LHCBM6 antibody cross reactivity. α-LHCII and α-LHCBM6 antibodies were tested for their cross-reactivity against different LHCBM) and CP26 and CP29 subunits. Recombinant LHCBM1, LHCBM2, LHCBM3, LHCBM4, LHCBM5, LHCBM6, LHCBM9, CP26 and CP29 apoproteins were overexpressed in E. coli and purified as inclusion bodies. 7µg of each apoproteins were loaded on SDS-PAGE gel for wastern blot analysis. Panel A and C reports the Red ponceau staining of filter used for immunoblotting. Panel B and D report the result of immunoblotting analysis using the antibody α-LHCII (Panel B) and α-LHCBM6 (Panel D).

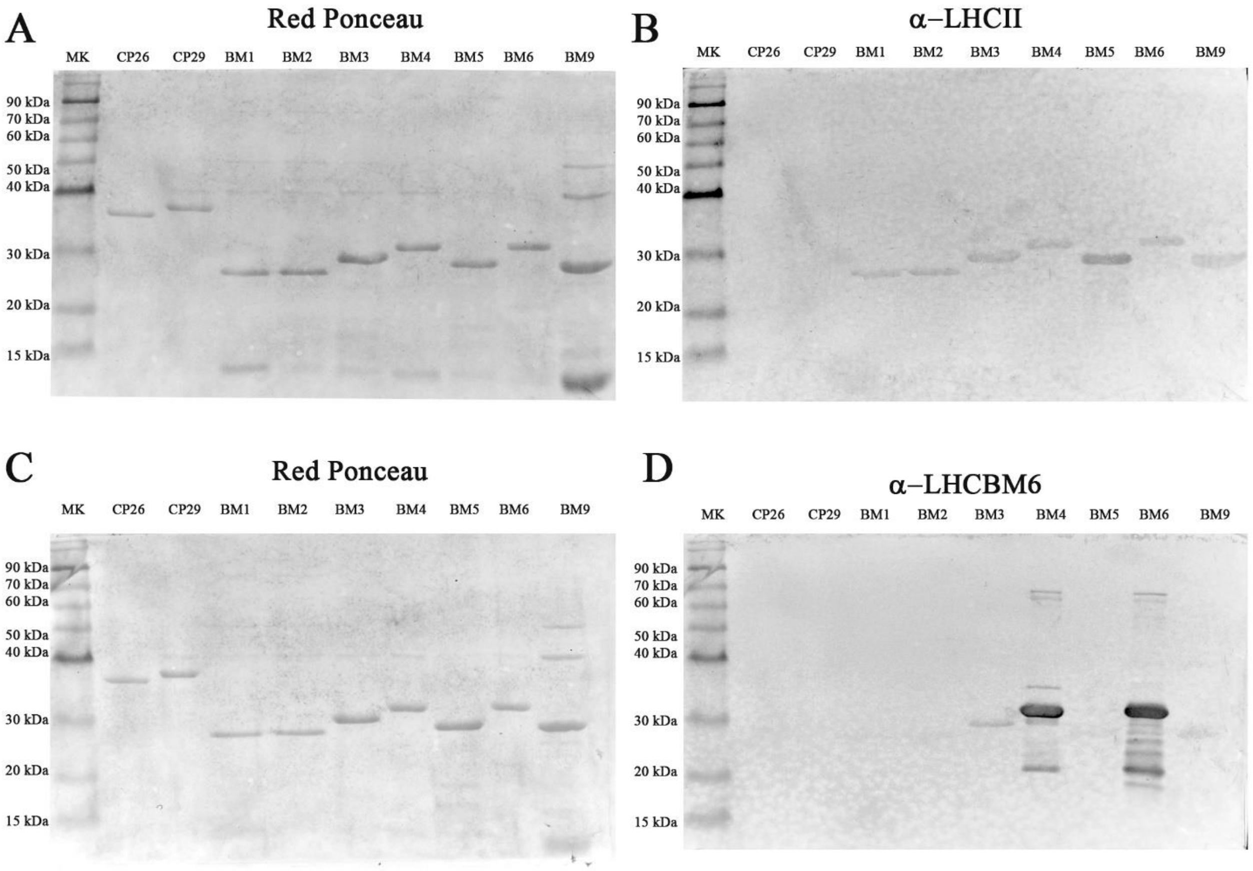

Figure S3 Determination of LHCBM4/6/8 abundance in thylakoid membrane. The amount of LHCBM4/6/8 in thylakoid membranes was evaluated by immunoblotting reactions using the recombinant LHCBM4 or LHCBM6 proteins as reference. For comparison the same procedure was applied for LHCBM1 and total LHCII trimers using recombinant LHCBM1 and native LHCII purified from thylakoid membranes as reference. Panel A: immunoblotting reactions with the indication of the µg of chlorophylls (Chls) loaded in each lane). Panel B: amount of LHCII, LHCBM1 and LHCBM4/6/8 in thylakoid membranes expressed as the ratio between µg of Chls bound by LHC proteins per µg of Chls in thylakoid membranes. The determination of LHCBM4/6/8 amount was calculated using LHCBM4 ([a]) or LHCBM6 ([b]) as reference. Error bars indicate standard deviation (n=3).

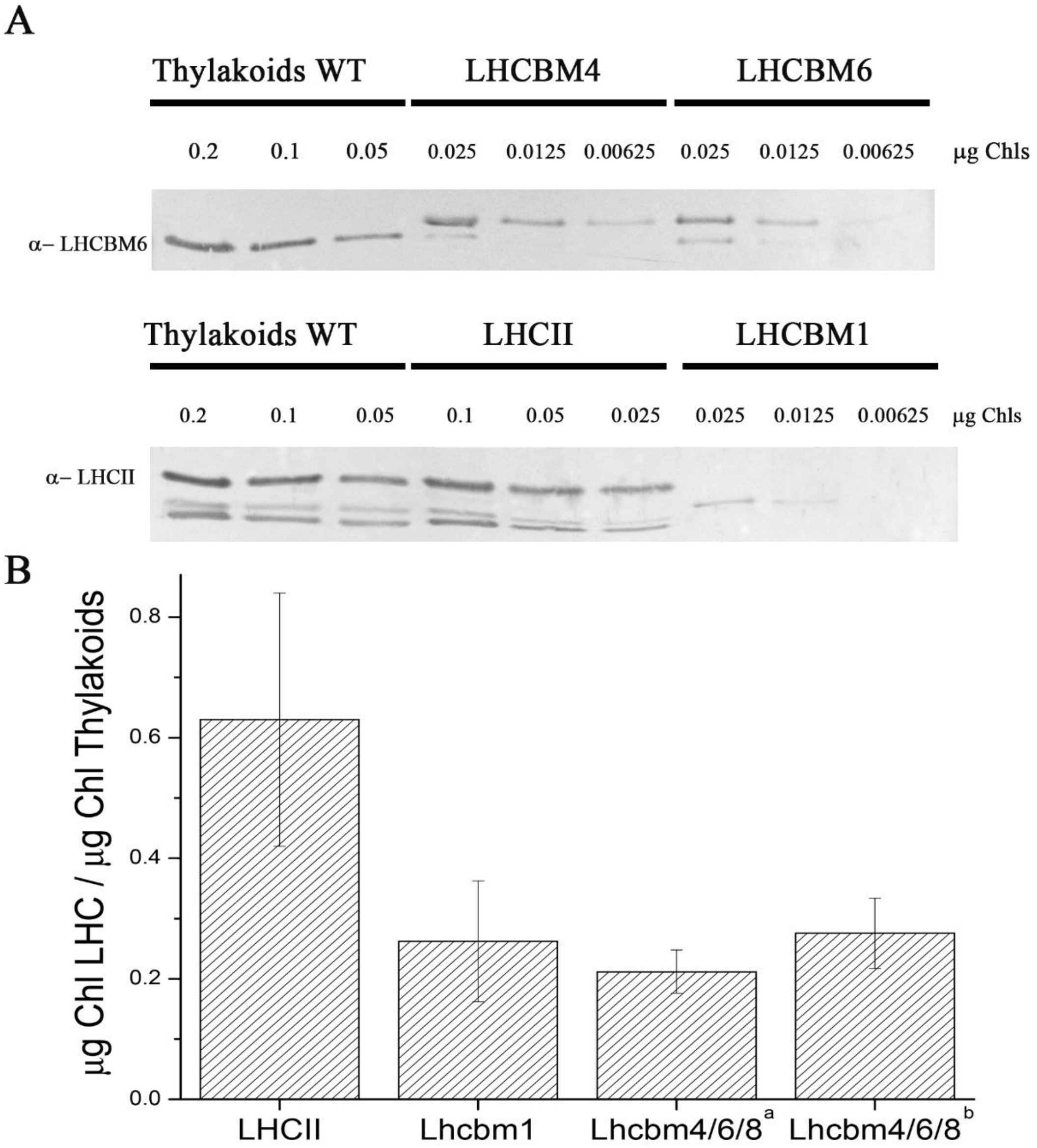

Figure S4 - Schematic maps of the constructs used. A) Silencing cassette in the pChlamyRNA3 vector. This vector was engineered to express a 21 nucleotide silencing RNA. The PSAD promoter and terminator control the rate of amiRNA transcription. Two amiRNAs (LCBM6A and LHCBM6B) were designed to silence LHCBM6 gene. One amiRNA (LHCBM4+6+8) of the four designed was effective in triggering silencing of LHCBM4, LHCBM6 and LHCBM8 genes. B), C) and D) Target regions of the amiRNAs on LHCBM4 (Panel B), LHCBM6 (Panel C) and LHCBM8 mRNAs (Panel D). For details on the amiRNAs sequence and features, see Table S1.

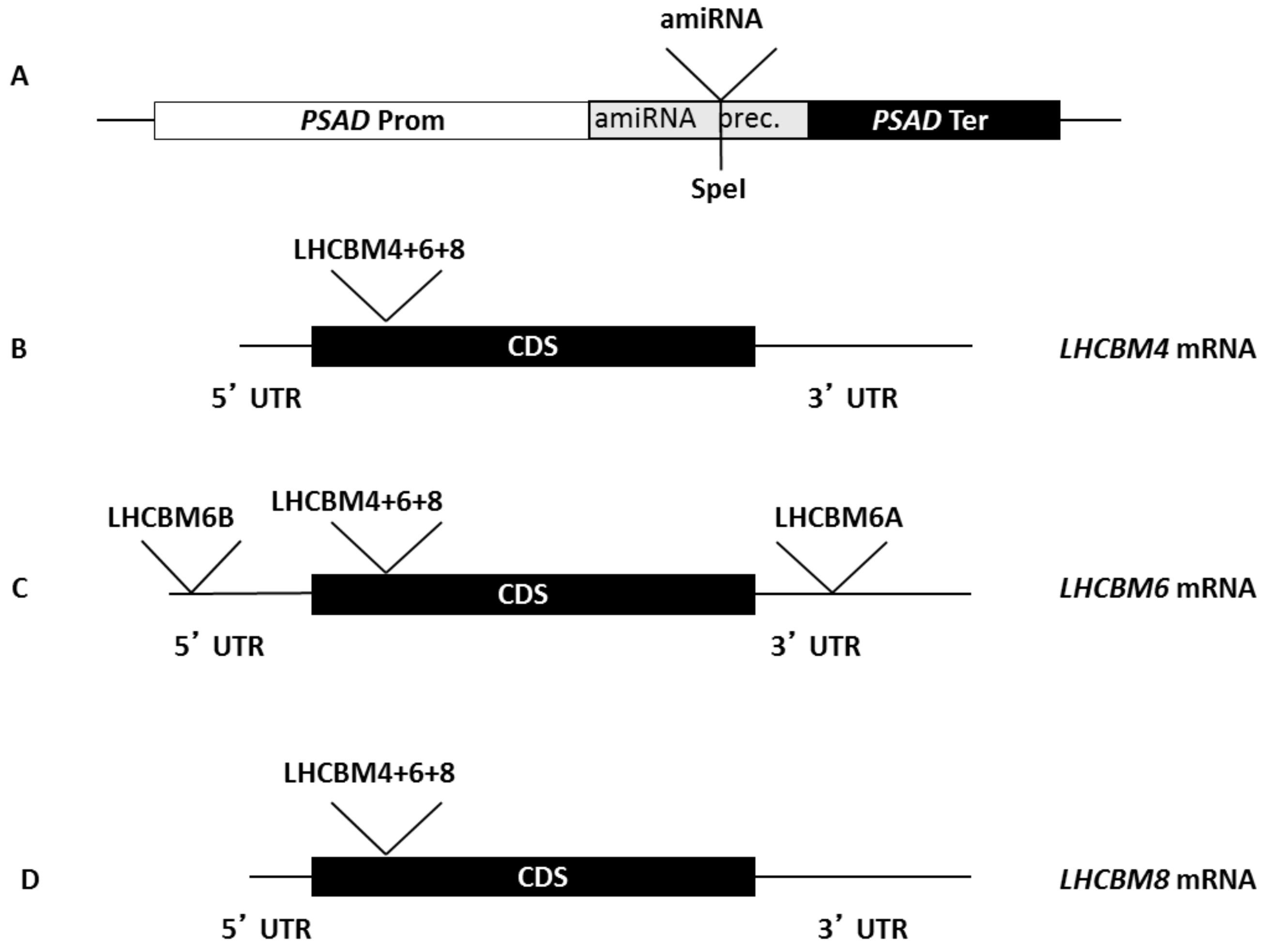

Figure S5: Determination of LHCBM mRNA level in WT and knock down strains. mRNA level was quantified through quantitative Real Time RT-PCR on RNA extracts from cells of WT and knock down strains grown in minimal medium (HS) in control light condition. The amount of LHCBM mRNA level is expressed as a ratio with the mRNA of RBCS2 mRNA (ribulose -1, 5 - bisphosphate carboxylase/oxygenase small subunit 1 gene).

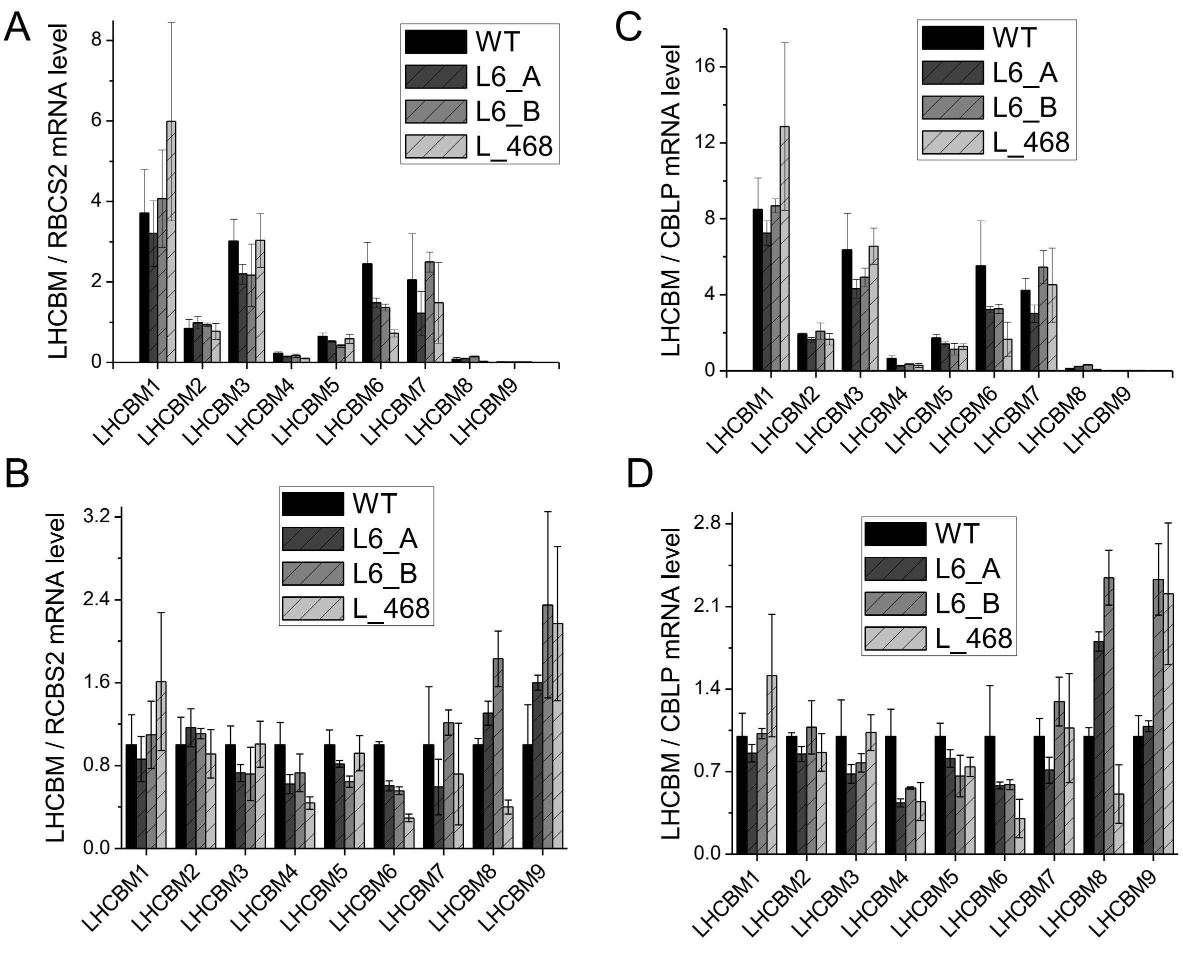

Figure S6. Chlorophylls, Photosystems and LHC distribution in 2D-PAGE. The distribution of chlorophylls in CN-PAGE as Integrated Optical Density (IOD) is reported on the top of the figure. The distribution of immunoblot signal of PsaA, CP43, LHCII and LHCBM4/6/8 on 2D-PAGE is reported as IOD. The main composition of CN-PAGE spot is indicated on the base of immunoblot results.

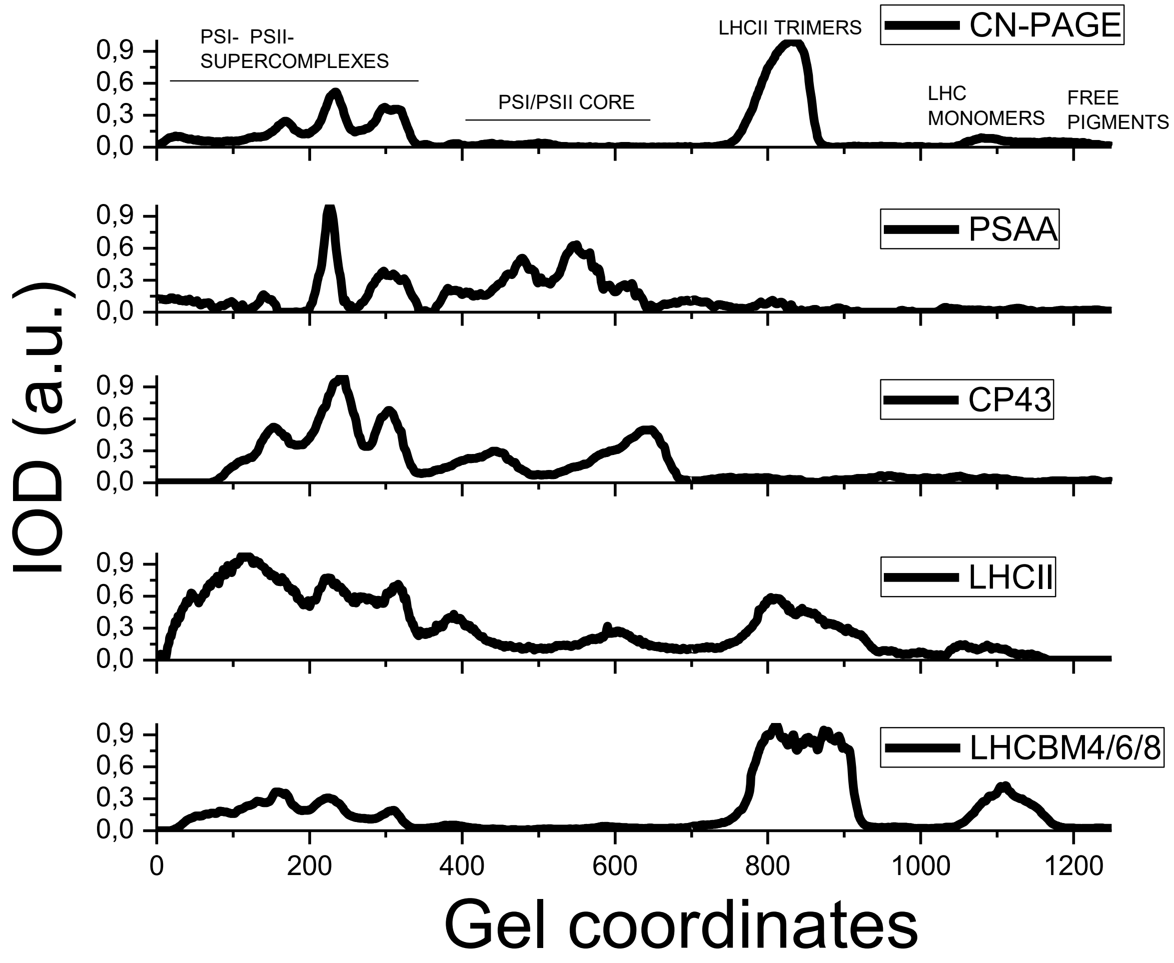

Figure S7. Fluorescence emission spectra at 77K of whole cells induced to state 1 or state 2. C. reinhardtii cells were induced to state 1 or state 2 as described in the methods section. The 77K florescence emission in state 1 and stat2 were normalized to the 686 nm peak, related to PSII emission. The stt7 mutant was included as negative control.

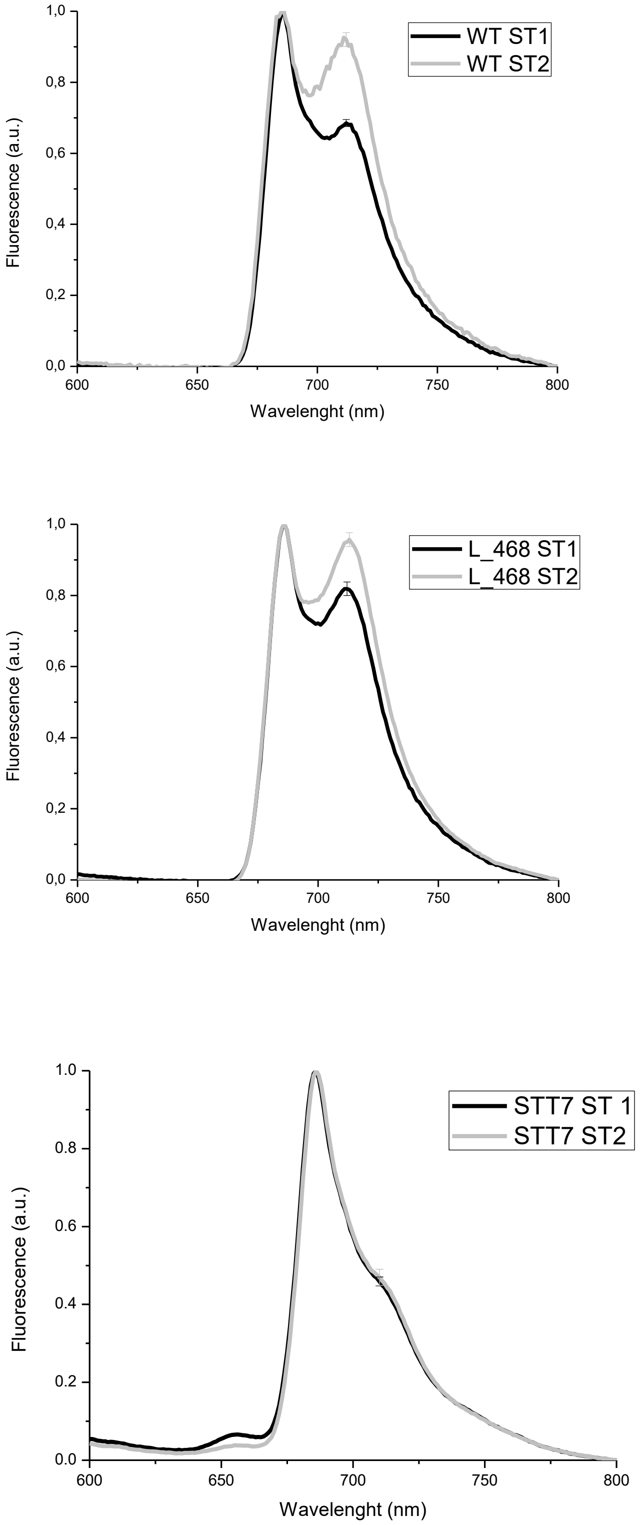

Figure S8. Correlation of NPQ values with LHCBM4/6/8, LHCBM1 or LHCSR content per PSII. NPQ values measured for WT and silencing strains reported in Figure 8C were plotted as function of LHCBM4-6-8 (Panel A), LHCBM1 (Panel B) or LHCSR (Panel C) content per PSII calculated on the base of the western blot analysis reported in Figure 8D-E. Linear regression is reported for Panel A data, with Adjusted R^2 value of 0.84. Linear regression for data reported in Panel B or C was not successful with Adjusted R^2 values of 0.62 and -0.19 respectively.

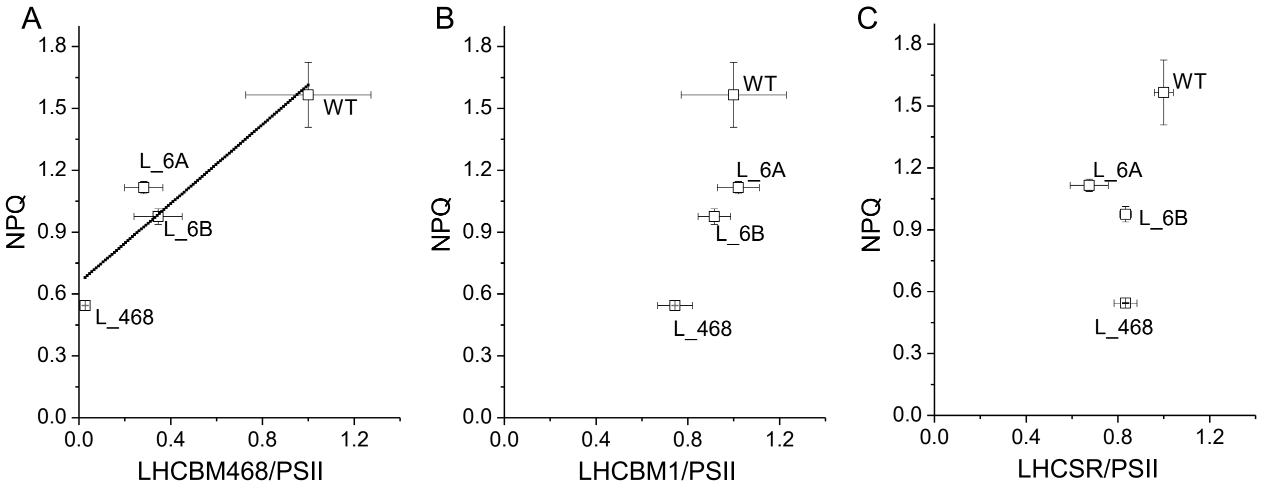

Figure S9. Analysis of the distribution of LHCBM4/6/8 and LHCSR3 protein in the thylakoids membrane by 2-D electrophoresis and immunoblotting. Thylakoid membranes of knock-down strains acclimated to high light (400 µmol $m^{-2}s^{-1}$), were solubilized with 1% of dodecyl-maltoside (α-DM) and separated by a Clear-Native PAGE (CN-PAGE) followed by a second dimension separation by SDS-PAGE. Immunoblot detections of LHCSR (antibody α-LHCSR), LHCBM4/6/8 (antibody α-LHCBM6), PSI and PSII (antibody α-CP43) are reported.

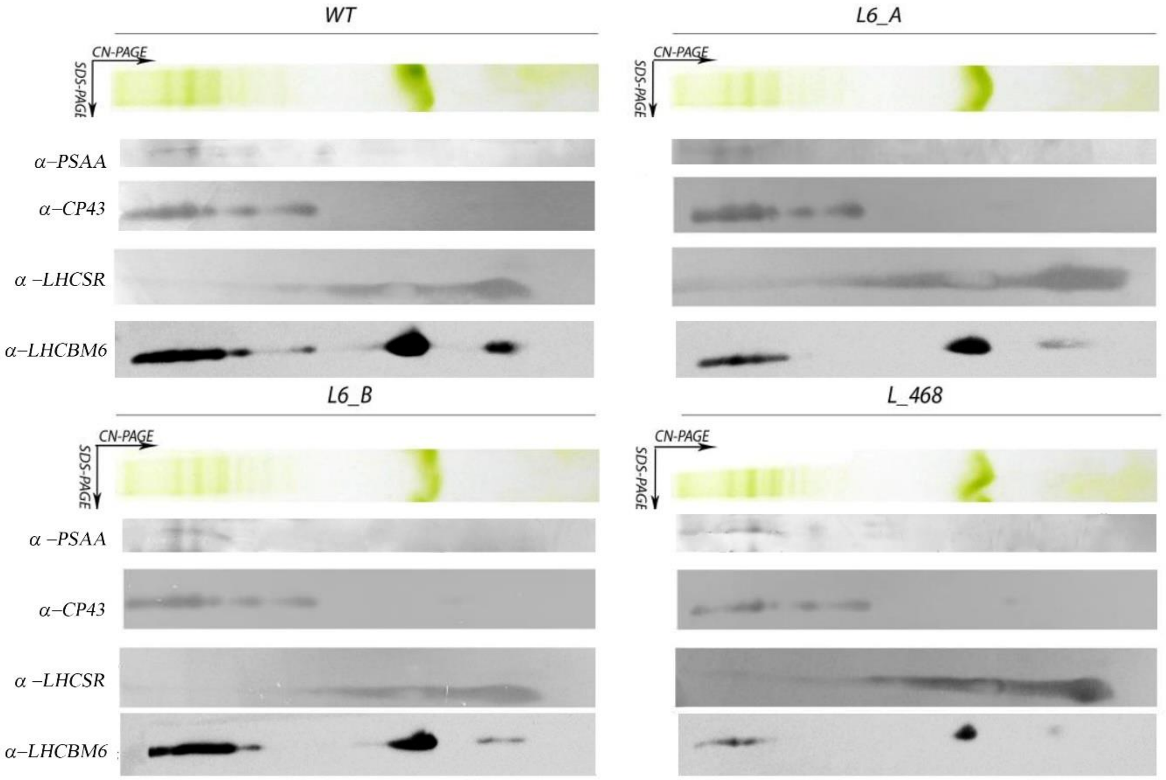

Supplement: Supplementary Data [file erw462_Supplementary_Data.zip › supplementary_tables_S1_S2_figures_S1_S9.pdf]
